# Antibiotic resistance gene sequencing is necessary to reveal the complex dynamics of immigration from sewers to activated sludge

**DOI:** 10.3389/fmicb.2023.1155956

**Published:** 2023-04-26

**Authors:** Claire Gibson, Susanne A. Kraemer, Natalia Klimova, Bing Guo, Dominic Frigon

**Affiliations:** ^1^Department of Civil Engineering and Applied Mechanics, McGill University, Montreal, QC, Canada; ^2^Aquatic Contaminants Research Division, Environment and Climate Change Canada, Montreal, QC, Canada; ^3^Department of Civil and Environmental Engineering, Centre for Environmental Health and Engineering, University of Surrey, Surrey, United Kingdom

**Keywords:** immigration, community coalescence, antimicrobial resistance, wastewater, activated sludge, community assembly

## Abstract

Microbial community composition has increasingly emerged as a key determinant of antibiotic resistance gene (ARG) content. However, in activated sludge wastewater treatment plants (AS-WWTPs), a comprehensive understanding of the microbial community assembly process and its impact on the persistence of antimicrobial resistance (AMR) remains elusive. An important part of this process is the immigration dynamics (or community coalescence) between the influent and activated sludge. While the influent wastewater contains a plethora of ARGs, the persistence of a given ARG depends initially on the immigration success of the carrying population, and the possible horizontal transfer to indigenously resident populations of the WWTP. The current study utilized controlled manipulative experiments that decoupled the influent wastewater composition from the influent microbial populations to reveal the fundamental mechanisms involved in ARG immigration between sewers and AS-WWTP. A novel multiplexed amplicon sequencing approach was used to track different ARG sequence variants across the immigration interface, and droplet digital PCR was used to quantify the impact of immigration on the abundance of the targeted ARGs. Immigration caused an increase in the abundance of over 70 % of the quantified ARGs. However, monitoring of ARG amplicon sequence variants (ARG-ASVs) at the immigration interface revealed various immigration patterns such as (i) suppression of the indigenous mixed liquor ARG-ASV by the immigrant, or conversely (ii) complete immigration failure of the influent ARG-ASV. These immigration profiles are reported for the first time here and highlight the crucial information that can be gained using our novel multiplex amplicon sequencing techniques. Future studies aiming to reduce AMR in WWTPs should consider the impact of influent immigration in process optimisation and design.

## 1. Introduction

Antimicrobial resistance (AMR) is recognized as one of the greatest threats to public health worldwide (Abadii et al., 2019). Each year 700,000 deaths are attributed to AMR and without action this number is predicted to rise to 10 million by 2050 (O’Neill, 2014). The United Nations Environment Assembly (UNEA-3) have recognized the importance of the environment in the development, spread and transmission of AMR to humans and animals (United Nations Environment Programme, 2022). Of particular interest are wastewater treatment plants (WWTPs), which have been identified as hotspots of AMR (Rizzo et al., 2013) and gateways to the environmental spread. Although a reduction in the load is observed, the wastewater treatment process does not effectively remove all phylogenetically mobile antibiotic resistance genes (ARGs) and resistant bacteria (ARB) before release into the environment (Lapara et al., 2011). Consequently, effluent wastewater has been shown to contribute to antibiotic resistance in surface waters and sediments downstream of effluent discharge points (Quintela-Baluja et al., 2019; Reichert et al., 2021). ARGs are also disseminated in the waste biosolids produced during the treatment process (Munir et al., 2011; Gao et al., 2012), which are often applied to agricultural land as fertilizers which creates another route of AMR dissemination. To minimize the spread of AMR, wastewater treatment plant design requires urgent optimization for the removal of ARB and ARGs.

With increasing knowledge of emerging contaminants in wastewater and the benefits of water resource recovery, the need for new wastewater treatment technologies is widely recognized. In this context, studies have aimed to minimize ARG release into the environment with novel design. However, results are often variable. For example, some studies of anoxic-aerobic membrane bioreactor observed a reduction in the abundance of ARGs (Le et al., 2018; Zhu et al., 2018), whilst others found the abundance of genes such as *tet*C to increase (Xia et al., 2012). The use of ozonation to reduce ARG loads resulted in removal efficiencies which varied between ARG classes (Staley et al., 2019). Similarly, some found chlorination to cause large reductions in the abundance of ARGs (Zhang et al., 2019) and increases in the abundance of cell free ARGs (Liu et al., 2018), whilst others reported no impact on genes such as *bla*TEM-1 (Pang et al., 2016). These contradictory results exemplify our lack of understanding of the drivers in the persistence and proliferation of ARBs and ARGs in WWTPs, which remains one of the greatest hurdles in developing appropriate treatment strategies to reduce AMR.

Influent wastewater contains a plethora of ARB which harbor several ARGs with specific amplicon sequence variants (ASVs) often associated with the genetic context of the gene (Gibson et al., 2023b). Each ARG variant is likely to obey different elimination or persistence mechanisms following their immigration into the biological treatment process from the sewer. The characterization of these mechanisms, however, requires the identification and quantification of ARG amplicon sequence variants across the influent and activated sludge (AS-WWTP) interface with high resolutions and sensitivity (Smith et al., 2022; Gibson et al., 2023b). Although quantitative PCR is sensitive, it is uninformative with regards to ARG sequence variants. Conversely, metagenomic shotgun sequencing can provide information on the genetic context of the most abundant variants, but it has limited capabilities in the identification of variants occurring in low abundance, and is less sensitive than qPCR for ARG detection (Bharti and Grimm, 2021). In environmental samples such as soil and activated sludge, metagenomic sequencing depth is insufficient to represent all diversity within these complex communities (Zaheer et al., 2018; Jankowski et al., 2022). Numerous studies have used data on ARG occurrence in the influent and activated sludge to infer the origin of AMR. However, more recent studies demonstrate that ARGs can vary at the sequence level based upon their origin (Zhang et al., 2021). The development of specific approaches to address this knowledge gap is the main goal of the current study.

The fate of immigrating ARB and ARGs is related to the complex ecological dynamics occurring at the interface between the influent and AS-WWTP. Firstly, the persistence of a given ARG depends on the immigration and success of its carrying population in the downstream community (Gibson et al., 2023a). Some ARGs may persist because the carrying population is already an indigenous resident of the AS-WWTP community. In this case, the ARG may not even be present in the influent community. Secondly, the persistence of a given ARG may also be impacted by its phylogenetic mobility. Horizontal gene transfer has been demonstrated to play a pivotal role in the spread of ARG across species (Von Wintersdorff et al., 2016). Although a given ARB may be unsuccessful in the AS-WWTP community, processes such as conjugation, transformation and transduction enable the movement of ARGs and increase the likelihood of ARG persistence by transferring to other, better adapted, hosts. For example, conjugative plasmids have been shown to play a significant role in facilitating the persistence of multidrug resistant ARB in WWTPs (Che et al., 2019). To determine the contributions of these mechanisms, ARG amplicon sequence variants (ARG-ASVs) need to be tracked across the immigration interface between the influent wastewater and the activated sludge. The current study employed a novel multiplex amplicon sequencing approach (Smith et al., 2022; Gibson et al., 2023b) to study ARG amplicon sequence variant dynamics with the required sensitivity and resolution. Such a technique will likely provide valuable insights into the contradictory results on the persistence of ARGs.

The compounded impact of complex immigration dynamics, community composition drifts and horizontal gene transfers on the fate of ARGs are better studied in highly controlled and replicated reactor experiments. In full-scale WWTP, the influent substrate compositions, bacterial population, and ARGs vary on a daily basis (Guo et al., 2019; Sun et al., 2021). These variations, even when small, limit our ability to accurately assess the exact mechanisms behind influent immigration and AMR in AS-WWTPs. Therefore, this study utilized triplicated lab-scale reactors fed with a synthetic wastewater, which allowed for a high level of control on the immigration process and the possibility of reproducing similar conditions in subsequent manipulative experiments. Among the operated reactors, the only varying factor was immigration, which was simulated by the addition of naturally occurring microbial communities (i.e., suspended solids) harvested in municipal wastewaters to the synthetic wastewater.

The ARG analyses presented here expand upon previous observations on the population dynamics and community assembly obtained with the same experimental set-up and published previously (Gibson et al., 2023a). Droplet digital PCR was used to quantify the variation in abundance of the ARGs across the immigration interface. Due to influent wastewater containing ARGs originating from several sources [e.g., clinical waste, sewer biofilms, and agricultural runoff (Guo et al., 2019; Qin et al., 2020)], it was hypothesized that ARG-ASVs from the influent wastewater would be different than the ones occurring endogenously in the activate sludge. Thus, multiplex amplicon sequencing was used for ARG source tracking.

## 2. Materials and methods

### 2.1. Samples

**FIGURE 1:**
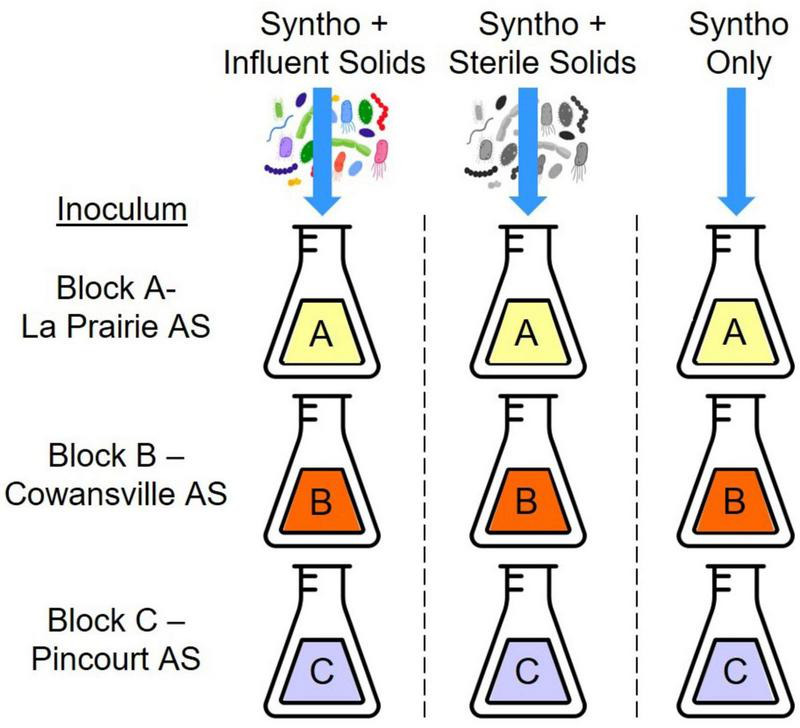
One set of reactors which received one source of influent solids (27 reactors). Reactors were inoculated with three different activated sludge communities (Block A-C). One third of the reactors received synthetic wastewater (Syntho) supplemented with influent solid which was the group with immigration (9 reactors). Another third of the reactors received Syntho and sterile solids which acted as a substrate control (9 reactors). The final third of reactors received Syntho only and acted as a continuity control (9 reactors).

Samples for analysis were collected from reactors previously operated as described by Gibson et al. (2023a). Briefly, small scale activated sludge (AS) reactors were inoculated with mixed liquor taken from three full scale AS-WWTPs (Figure 1: Block A-La Prairie AS, Block B-Cowansville AS, and Block C-Pincourt AS). Reactors were operated with a hydraulic retention time of 1.8 days and a solid retention time of 5 days, and fed with a synthetic wastewater (Syntho; Boeije et al., 1999). A synthetic wastewater was utilized to ensure a stable wastewater composition was maintained over time, this could not be achieved using wastewater obtained from a full-scale WWTP which is impacted by numerous factors. Synthetic wastewater (Syntho) was produced in the laboratory using the recipe developed by Boeije et al. (1999) and autoclaved at 121°C and 5 PSI for 30 min to sterilize. The main carbon sources within Syntho were sodium acetate, starch, glycerol, and milk powder. During Phase 1 of reactor operation, all reactors received Syntho only to ensure that a stable community was formed and any existing immigrants were removed. In Phase 2, to investigate the impact of immigration, in one third of the reactors the synthetic wastewater was supplemented with influent solids (i.e., an influent wastewater community) taken from a full-scale AS-WWTP. Two control groups were also included, the first was a substrate control, that received Syntho supplemented with sterile influent solids (autoclaved at 121°C, 15 psi for 30 min). The final nine reactors received Syntho only to act as a continuity control. Finally, during Phase 3 of reactor operation, influent solids were removed from the feeds to determine if the impact of immigration was maintained over time.

This experiment was repeated with the use of three different sources of influent wastewater community. It was observed that the impact of immigration was similar in each set of reactors, thus only one set of reactors was selected for ARG analysis (27 reactors). The chosen reactors received influent solids from Cowansville AS-WWTP (Set B; Gibson et al., 2023a). For ARG analysis, samples from each Block (with the same inoculum; Block A-La Prairie AS, Block B-Cowansville AS and Block C-Pincourt AS), receiving the same feed were pooled (three biological replicates pooled). A total of 12 samples collected during Phase 2 of reactor operation were analyzed: three groups with immigration, three groups with sterilized influent solids, three with synthetic wastewater only, and three influent wastewater samples as shown in Figure 1.

### 2.2. Sample collection and processing

Mixed liquor biomass samples were collected from the reactors and centrifuged in 2 mL microcentrifuge tubes for 5 min at 16,000 × *g*. The supernatant was discarded and biomass was stored immediately at -80^°^C until nucleic acid extraction (approximately 3 months). Three aliquots were stored per reactor sample. DNA was extracted from approximately 0.25 g of each stored biomass sample using DNeasy PowerSoil Kit (Qiagen, Germantown, MD, USA) following the manufacturer’s instructions with a final elution volume of 100 μl. One blank was included per batch of extractions (approximately 24 samples). Samples were extracted in duplicate, and DNA aliquots were immediately stored at -80^°^C until future use.

The quality of nucleic acids was assessed using the ratio of absorbance at 260 nm and 280 nm (260/280) obtained using NanoDrop™ One. DNA extracts with a 260/280 value between 1.8 and 2.0 were considered to have good purity. Quant-iT™ PicoGreen™ dsDNA Assay kit (Invitrogen, ON, Canada) was used to accurately quantify the double-stranded DNA concentration of the extracts prior to droplet digital PCR.

### 2.3. Droplet digital PCR

A total of 15 different antibiotic resistance genes were analyzed using droplet digital PCR and multiplexed amplicon sequencing (Supplementary Table 1). Clinically relevant ARGs were selected which displayed resistance to five classes of antimicrobials; beta-lactams (*bla*MOX, *bla*TEM, *bla*OXA), fluoroquinolones (*qnr*S, *qnr*B), diaminopyrimidines (*dfr*A), macrolides (*mph*E, *ere*A) and tetracyclines (*tet*O, *tet*Q, *tet*E) to investigate whether the impact of immigration varied between ARG classes. In addition, four multidrug resistance genes were included (*rob*A, *msr*D, *qac*L, and *mar*R). Primers were designed and *in silico* verification was conducted by Swift Biosciences, Ann Arbor and manufactured by Integrated DNA Technologies, USA. Appropriate DNA dilutions were determined using a standard quantitative PCR reaction (Powerup SYBR green master mix) performed using three DNA dilutions to check for PCR inhibition. The fold dilution for digital droplet PCR was calculated using *Eq*.1. Samples were diluted to the appropriate concentration using DNase/RNase free water.


(1) $$D\,i\,l\,u\,t\,i\,o\,n\,\left(f\,o\,l\,d\right)=2^{24-C\,q}$$

A 20 μl reaction mix was prepared for each droplet digital PCR reaction, which included 1 × QX200™ ddPCR™ Evagreen^®^ Supermix (Bio-Rad, CA, USA; catalogue number 1864033), 100 nM of the forward and reverse primers, template DNA as determined using equation 1, and RNase/DNase-free water. For quality control purposes, a positive control obtained from wastewater samples was used in each run to ensure the matrix was representative of the actual samples to be analyzed. In addition, a no template control was included. Droplets were generated using the Bio-Rad QX200™ Droplet Generator and DG8™ Cartridges with 20 μl of reaction mix and 70 μl of Droplet Generation Oil for Evagreen. Following droplet generation, droplets were transferred to a 96 well plate and sealed with foil using a Bio-Rad PX1 PCR Plate Sealer. A Bio-Rad C100 Touch Thermal Cycler was used for thermal cycling of all plates. Reaction conditions used were as follows; 95°C for 5 min., followed by 50 cycles of 95°C for 30 s., *x*°C for 60 s, and 72°C for 30 s. whereby *x* is the annealing temperature (Supplementary Table 1). Followed by 4°C for 5 min, 90°C for 5 min, and a hold at 12°C. Annealing temperatures were optimized using a gradient PCR method on a pooled DNA sample. The optimal annealing temperature was selected based upon the separation between positive and negative droplets, and the lowest “rain” i.e., droplets defined as neither positive or negative. The primer concentration was optimized by conducting a test assay on a representative pooled DNA sample, at various primer concentrations ranging from 100 to 250 nM. A final primer concentration of 100 nm was selected for the assays. To test for inhibitor and matrix effects, 10-fold dilutions of pooled DNA samples were quantified.

Droplets were analyzed using the Biorad QX200™ Droplet Reader and the absolute quantification (ABS) experimental setup. Results were visualized using the QuantaSoft™ Software (Bio-Rad, version 1.7.4) to provide an absolute quantification of the ARG per nanogram of DNA. Reactions producing fewer than 10,000 droplets were excluded and the droplet digital PCR reaction was repeated. The threshold was defined using the auto-select function in the QuantaSoft™ software (Bio-Rad, version 1.7.4). In cases where an appropriate threshold was not automatically selected, a threshold was manually chosen. An example of a positive and negative result are provided in Supplementary Figure 1A. All samples were analyzed in duplicate in different PCR runs to confirm the technical reproducibility. Copies of each ARG/ng-DNA were normalized to the copies of 16S rRNA gene/ng-DNA to provide a relative ARG copy number/16S rRNA gene. The limit of blank (LOB) was calculated using Eqs 2, 3 (Armbruster and Pry, 2008) was used to determine the limit of detection (LOD) of samples. To confirm the calculated LOD values, assays were performed using six dilutions of a positive sample (Supplementary Figure 1B), and the 95% LOD was determined based upon the probability of detection at each concentration.


(2) $$L\,O\,B=M\,e\,a\,n_{B\,l\,a\,n\,k}+1.645\,\left(S\,D_{B\,l\,a\,n\,k}\right)$$

(3) $$L\,O\,D=L\,O\,B+1.645\,\left(S\,D_{L\,o\,w\,e\,s\,t\,s\,a\,m\,p\,l\,e}\right)$$

### 2.4. Sequence diversity of ARGs

A multiplexed amplicon sequencing approach was used to analyze sequence variant diversity among the ARGs detected in the reactors. ARGs were amplified using a multiplex PCR kit (Qiagen) with the following reaction conditions; 95°C for 15 min. followed by 25 cycles of 94°C for 30 s, 60°C for 90 s, and 72°C for 60 s, and a final extension step at 60°C for 30 min. Amplicons obtained from individual PCR reactions (using one set of primers per reaction) were pooled and used as a positive control. The products of the multiplex PCR reaction were purified using SPRI Select beads (Beckman Coulter) to remove primer dimers at a sample to bead ratio of 0.8. Samples were barcoded and pooled at equimolar concentration. The pooled samples were sequenced on the Illumina MiSeq PE250 platform at McGill University and Génome Québec Innovation Centre (Montréal, QC, Canada).

ARG Amplicon sequence variants (ARG-ASVs) were defined as sequences which differed by at least one single nucleotide variant (SNP) within the amplified region of the targeted gene (around 275 base pairs). To distinguish between sequencing errors and actual sequence variants, a stringent filtering process was applied as described in Section “2.5. Bioinformatics.”

### 2.5. Bioinformatics

Reads from each sample were split according to their forward and reverse primer sequences allowing for one sequencing error per primer sequence using a custom script available upon request. Read pairs where both or one of the reads was missing a primer sequence at the beginning or where the primer sequences found did not correspond to each other were removed. Subsequently, we used Trimmomatic v. 0.39 to trim all reads using the following settings: LEADING: 3 TRAILING: 3, SLIDINGWINDOW:4:15 and MINLEN:36. Then we used vsearch v2.13.3 to merge the trimmed read pairs before converting them to fasta format. The merged reads where then sorted and clustered with an identity of 1 and a minimum length of 100 bases while keeping track of the cluster size using the -size out option.

PCR and sequencing errors created many unique or very low count amplicon sequence variants that we removed using a custom R script. First, we tested if the distribution of read counts for each amplicon sequence variant produced by an individual primer pair followed a Poisson distribution (centered around the mean of all counts) and corrected the resulting *p*-values (subtracted from 1 such that amplicon sequence variants with high read counts had low *p*-values) using the Bonferroni correction. If none of the amplicon sequence variant have a corrected *p*-value below 0.5, we removed the whole dataset, as this indicated that we are not able to distinguish real amplicon sequence variants from those generated by PCR or sequencing errors (noise sequence variants). If any amplicon sequence variants survived the filtering, we aimed to remove the noise sequence variants from the datasets by finding the minimum of a density function based on the total data for each gene (including noise sequence variants) and removing all amplicon sequence variants with counts less than the minimum found. By comparing with other filtering approaches [e.g., DADA2 (Callahan et al., 2016) or minimum absolute value], it was found that the filtering adopted here was the most stringent, but was still able to recover the diversity of synthetic mixes of sequence variants.

We subsequently combined all reads corresponding to a specific resistance gene (including multiple primers amplifying the same gene). Files were re-replicated using a custom python script available upon request (using the Pandas module), before constructing a table of allelic distribution across samples equivalent to an OTU table for each resistance gene using vsearch v2.13.3’s cluster_fast option with the output option. Unique consensus sequence variants were obtained using the centroids option. To ensure that the resulting amplicon sequence variants were the products of amplification of the target ARG, we blasted all consensus sequences to a custom ARG database using the diamond (v0.9.32.133) blastx option with an e-value of 3 and a minimum identity of 80. Consensus sequences that were not similar to any entries in the ARG database were removed from the analysis.

### 2.6. Statistics

The Mann-Whitney U test was used to determine if there was a statistically significant increase in the abundance of each ARG with immigration. Samples taken from the reactors with immigration (receiving Syntho and influent solids) were compared to the control groups at a significance level of *p* < 0.05.

Procrustes analysis was performed using the R Vegan package (Oksanen et al., 2022) to determine whether the microbial community of the reactors correlated with the ARG profile. Procrustes was used to compare the ARG and microbial community ordinations. ARG data was standardized to means of 0 and standard deviation of 1. Using the R Vegan package, Principal Component Analysis (PCA) was conducted for the ARG dataset based on the Euclidean distances. Principle coordinate analysis (PCoA) was performed using Jaccard distance on the microbial community data, as this distance was previously shown to most effectively display the impact of immigration on the community in the current experiment (Gibson et al., 2023a). The function “Protest” was used with 999 permutations to test the significance between the configurations.

### 2.7. Database analysis of amplicon sequence variants

The NCBI database (NCBI Research Coordinators, 2013) was used to search for previously reported hosts of the detected ASVs. To evaluate the mobility of each ASV, PLSDB (Galata et al., 2019) was used to determine whether they had been previously reported within plasmids. In this analysis, only 100% identity and coverage results were considered as ASVs could vary by as little as a single SNP.

## 3. Results

### 3.1. Impact of immigration on the abundance of ARGs in the activated sludge

Reactors were operated under highly controlled conditions to test the impact of immigration on antibiotic resistance in the activated sludge. All 27 reactors in the three Blocks (three different inoculum) received a synthetic wastewater (Syntho) feed, which allowed the influent wastewater composition to be carefully controlled throughout reactor operation. To ensure the reactors were operating at steady state, the chemical oxygen demand and suspended solids concentrations were monitored over time and observed to be relatively constant (steady-state). After operating the reactors for 12 SRTs and ensuring steady state was reached, during Phase 2 in each Block (nine reactors in total; Block A-La Prairie AS, Block B-Cowansville AS, and Block C- Pincourt AS), three reactors received synthetic wastewater with added influent solids to simulate the impact of immigration. Another three of the reactors received autoclaved influent solids and acted as a substrate control. The final three reactors received Syntho only as a continuity control to establish a baseline for the level of AMR without immigration.

The impact of influent immigration on the activated sludge microbial community is discussed at length in Gibson et al. (2023a). Briefly, immigration impacted the microbial community composition of the activated sludge, and reactors with immigration became more similar to the microbial community of the influent solids. Up to 25% of sequencing reads were observed to be contributed through influent immigration, representing a significant proportion of the activated sludge community. Using a mass balance approach, it was observed that the growing immigrant population typically exhibited a lower and often negative net growth rates in the activated sludge, when compared to the core resident genera which typically displayed a positive net growth rate. In Gibson et al. (2023a), focus was placed on the impact of immigration on the microbial community composition of the activated sludge alone, whilst in this publication the impact on AMR is explored.

**FIGURE 2:**
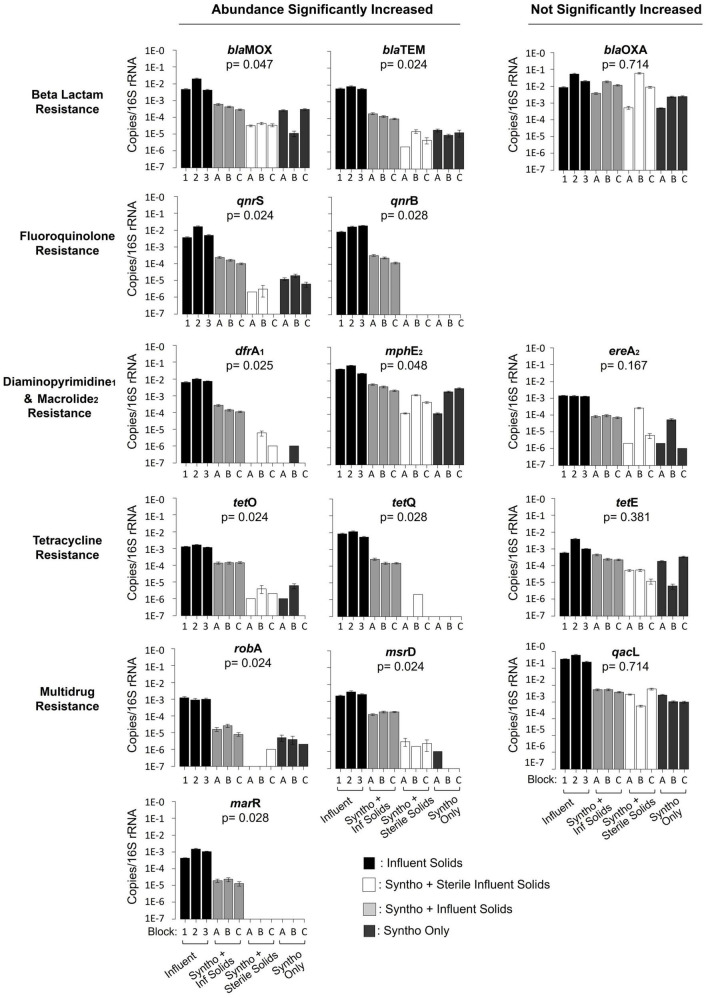
Quantification of antibiotic resistance genes using droplet digital PCR. Reactors with immigration received Syntho and influent solids. Reactors receiving Syntho and sterile influent solids acted as a substrate control. Reactors receiving Syntho only acted as a no immigration continuity control. The Block represents the inoculum source of the reactors; A- La Prairie ML, B- Cowansville ML, C- Pincourt ML, Influent samples are numbered based upon the order received by the reactors during Phase 2. Each bar represents a composite sample of three biological replicates. The Error bars represent the standard error between duplicates. The Mann-Whitney U Test was used to assess the statistical significance of the change in abundance of each ARG with immigration when compared to the sterile and no immigration control combined.

To determine the impact of immigration on AMR in the AS, digital droplet PCR was used to quantitatively assess the concentration of fifteen ARGs in the reactor samples and influent wastewater. Results showed that immigration caused a significant increase in the relative abundance of eleven of the fifteen ARGs in the activated sludge when compared to the control groups (sterile control and no immigration control together) (Figure 2; Mann–Whitney U Test; *p* < 0.05). Genes observed to increase in abundance were distributed in several classes of antimicrobial resistance such as ARGs against fluoroquinolones (*qnr*B and *qnr*S), beta-lactams (*bla*TEM and *bla*MOX), macrolides (*mph*E), diaminopyrimidines (*dfr*A) and tetracyclines (*tet*Q and *tet*O). Three out of four of the efflux pump-associated multi-antimicrobial resistance genes quantified showed a significant increase in concentration with immigration (*mar*R, *msr*D and *rob*A). Genes such as *bla*MOX were present in the reactors with and without immigration, whilst others such as *mar*R and *qnr*B were detected in the activated sludge mixed liquor only with immigration.

The relative abundances of over 70% (11/15) of quantified ARGs increased in the activated sludge with immigration. However, they typically remained lower than those in the influent solids. To determine the impact of immigration on the overall ARG load, the absolute ARG concentrations were calculated (Supplementary Table 2) using information on reactor operation including the volatile suspended solid concentration, yield of DNA/g-VSS, 16S rRNA gene/ng-DNA and gene abundance (16S rRNA gene) (Supplementary Table 4; Gibson et al., 2023a). It was observed that the load of genes such as *bla*OXA increased by 0.42 log gene copies/L between the influent and mixed liquor. Whilst others such as *rob*A reduced by 1.14 logs of gene copies/L between the influent and activated sludge. Consequently, despite the reduction in ARG relative abundance (copies/16S rRNA gene) between the influent solids and activated sludge, high absolute ARG concentrations remained.

### 3.2. Correlation between the microbial community composition and ARG profile

**FIGURE 3:**
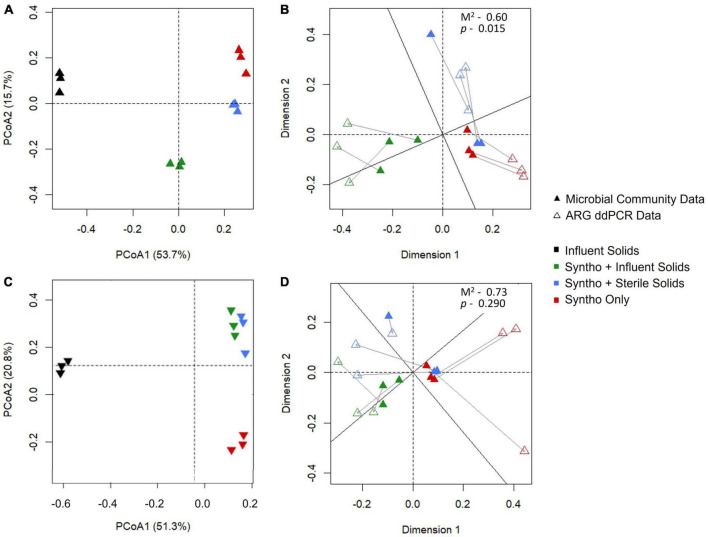
Procrustes analysis to investigate the correlation between the microbial community and ARG composition. **(A)** Microbial community of the reactors visualized using Jaccard dissimilarity as determined by 16S rRNA gene sequencing. **(B)** Procrustes analysis of microbial communtiy and ARG profile with Jaccard dissimilarity. **(C)** Microbial community of the reactors visualized using Bray Curtis dissimilarity as determined by 16S rRNA gene sequencing. **(D)** Procrustes analysis of microbial communtiy and ARG profile with Bray Curtis distance.

In numerous studies, the microbial community composition has increasingly emerged as a key determinant of ARG content (Forsberg et al., 2014; Wu et al., 2017; Zhou et al., 2017). In continuity with these observations, a Procrustes analysis was performed to determine whether the observed changes in the abundance of ARGs in the reactors correlated with changes in the microbial community composition with immigration. Procrustes analysis using the Jaccard distance matrix revealed a significant correlation (Procrustes *M*^2^ = 0.60, *p* = 0.015) between the microbial community compositions of the reactor activated sludge and the profiles and concentrations of ARGs they carried (Figure 3B). However, this correlation was not significant (Procrustes *M*^2^ = 0.73, *p* = 0.290; note that lower M^2^ means a better correlation between the datasets) when using the Bray-Curtis dissimilarity (Figure 3D).

### 3.3. Analysis of ARG amplicon sequence variants

**FIGURE 4:**
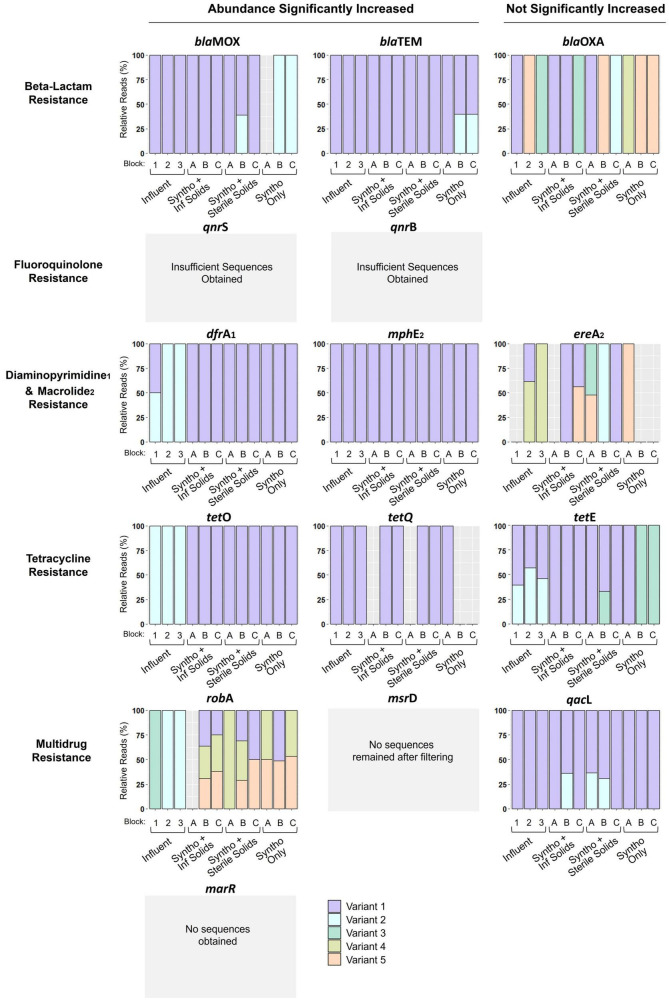
ARG ASVs detected using multiplexed amplicon sequencing. Reactors with immigration received Syntho and influent (Inf) solids. Reactors receiving Syntho and sterile influent solids acted as a substrate control. Reactors with Syntho only acted as a no immigration control. Each sample represents a pool of biological triplicates. Block represents the inoculum of the reactor A- La Prairie Mixed Liquor, B- Cowansville Mixed Liquor, C- Pincourt Mixed Liquor. Influent 1, 2, and 3 represent the order in which they were fed to the reactors during Phase 2, with influent 3 received for the final solid retention time (SRT). All reactor samples analyzed were obtained from the final day of Phase 2. The *msr*D, *mar*R, *qnr*B, and *qnr*S gene ASVs were undetected after the sequence filtering process either because they did not survive the stringent filtering process (*msr*D and *qnr*B), or insufficient reads were obtained resulting in no detection (*qnr*S and *mar*R).

Digital droplet PCR showed that the relative abundance of over 70% (11 out of 15) of the ARGs investigated significantly increased with immigration. However, questions remained about the exact dynamics occurring at the interface between the influent and activated sludge. Chief among them was whether the increase in abundance of an ARG was due to the introduction of genes originating from the influent. In other words, were the observed changes in the activated sludge likely due to direct ARG immigration or other changes induced by the presence of influent solids? To investigate the immigration dynamics at greater depth, a multiplexed amplicon sequencing approach was used to detect ARG-ASVs within the influent and reactor samples. By utilizing short read sequences, sequence diversity within a specific region of each ARG (around 275 base pairs) could be used as a marker to track the movement of genes between the influent and activated sludge. After stringent filtering based on the Poisson distribution, ARG-ASV information was obtained for eleven of the fifteen ARGs analyzed (Figure 4). The four remaining targets (*msr*D, *mar*R, *qnr*B, and *qnr*S) were undetected after the filtering process either because they did not survive the stringent filtering process (*msr*D and *qnr*B), or insufficient reads were obtained resulting in no detection (*qnr*S and *mar*R). Amplicon sequencing of the eleven ARGs revealed different ASV distributions or concurrence profiles between the influent and the activated sludge mixed liquors (Figure 4). In several cases, the ARG-ASVs were specific to either the influent or reactors, with few examples of concurrence in the reactors and the received influent solids. This demonstrates that PCR based methods over-simplified the ARG dynamics at the interface between the influent and activated sludge.

The concurrence profiles between the influent and the activated sludge reactors could be classified into 6 similarity groups that were typically confined either to the ARGs that significantly increased in abundance with influent immigration (Groups 1–3) or to ARGs with abundances that statistically remained unchanged (Groups 4–6; Figure 4). As summarized in Table 1, *Group 1* consists of ARGs for which the same and single ASV was observed in all samples (genes *mph*E and *tet*Q). This concurrence profile is consistent with direct immigration causing the significant increases in the abundances of these genes. *Group 2* collates ARGs with an ASV observed only in the control reactor samples, but not in the live immigration reactor or the influent samples (gene *bla*MOX and *bla*TEM). This concurrence profile is also consistent with direct immigration overwhelming the detection of the ARG-ASV observed in the control reactor. *Group 3* concurrence profiles are characterized by ARG-ASVs observed only in the influent, but not in the reactors (genes *dfr*A, *tet*O, *rob*A). This profile appears to follow a counter selection dynamic, whereby one or more of the influent sequence ASVs did not successfully immigrate between the influent and activated sludge. This demonstrated that the presence of a given ARG-ASV in the influent and the significant increase of an ARG with immigration, does not definitively predict the presence of the ASV within the activated sludge as could have been inferred using ddPCR results alone.

**TABLE 1:** Concurrence profiles of ARGASVs in the influent and reactor samples.

Group	Genes	Description	Conclusions
1	*mph*E, *tet*Q	Same single ASV observed in all samples	Consistent with direct immigration
2	*bla*MOX, *bla*TEM	Unique ASV present in control reactors that is not detected with immigration or in the influent solids	Direct immigration and suppression dynamics
3	*dfr*A, *tet*O, *rob*A	Unique ASVs present in the influent solids only	Counter Selection
4	*tet*E	Both 2 and 3: Unique ASVs present in the control groups and influent solids	Mixture of direct immigration, counter selection and suppression dynamics
5	*qac*L	Unique ASV only present in reactors receiving influent solids (live and autoclaved)	Possible direct immigration of ASVs occurring below detection limit
6	*bla*OXA, *ere*A	Numerous ASVs distributed throughout the influent and reactor samples	ASVs randomly distributed

The *Group 4* profile which was only observed for *tet*E appeared to be a mixture of Group 2 and 3 profiles, suggesting that these two mechanisms could be at interplay. Group 3 and 4 ARGs exemplify the complexity of interactions at the interface between the influent and activated sludge. Considering the Procrustes results (Figure 3), the increase in the abundances of these ARGs could be associated with the increase in relative abundance of the bacterial populations that carry these ARGs instead (Group 3) or simultaneously (Group 4) to direct ARG immigration.

The last two profiles of concurrences were observed for ARGs that did not significantly increase in abundance (Figure 4). *Group 5* profile was only observed for *qac*L and showed ASVs that were only observed in some samples from reactors receiving influent solids (both live and autoclaved). These ASVs were in low abundances in these samples. Thus, they may have been below the detection limit in the influent or control reactors, and immigration made them detectable in some circumstances possibly due to small variations in community compositions as in Groups 3 and 4. Finally, the genes of *Group 6* (*bla*_OXA_ and *ere*A) harbored 5 ASVs each present seemingly at random in samples from the influent or the reactors under different experimental conditions. Consequently, no clear profiles could be identified, and immigration did not appear to affect these genes.

### 3.4. Database information on the genetic context of ASVs

To investigate the genetic context of ARG-ASVs, the NCBI database and PLSDB (Galata et al., 2019) were utilized to gain information about previously reported hosts and gene occurrence on plasmids (Supplementary Table 3). To exemplify this approach and establish hypotheses for future work, genes from group 2–5 (Table 1) were analyzed, as unique ASVs were detected in either the reactors or influent samples.

In group two, analysis of the *bla*TEM ARG using the NCBI database revealed the ARG-ASVs to have previously been reported in similar bacterial hosts (Supplementary Table 3), which included taxa from the genus *Acinetobacter*, *Enterobacter*, and *Klebsiella* to name a few. The *bla*TEM ASV-1 was present in numerous variants of the *bla*TEM gene (over 60) including TEM-29, TEM-169, and TEM-26 whilst ASV-2 was present in only TEM-229, TEM-116, TEM-162, TEM-181, and TEM-157. Analysis of the two ARG-ASVs in PLSDB revealed both to have been previously reported on plasmids. Plasmids containing ARG-ASV 1 have been detected in numerous settings including in humans, animals and the environment (water). Whilst plasmids containing *bla*TEM ASV-2 have been reported in fewer settings, which included soil, feed additives and *Orcytes gigas* (rhinoceros beetle).

In group 3, analysis of the ASVs of the *dfr*A gene found that *dfr*A ASV-1 corresponded to the *dfr*A-5 gene, and ASV-2 the *dfr*A-14 variant of the gene (Alcock et al., 2020). ASV-1 (*dfr*A-5) and ASV-2 (*dfr*A-14) are phylogenetically closely related (Sánchez-Osuna et al., 2020) and are commonly observed within integrons and on plasmids. As with the *bla*TEM ASVs, both *dfr*A ASVs have been previously reported in similar host including taxa from the genus *Aeromomas, Enterobacter*, and *Citrobacter*.

In group 4, similar selection patterns were observed with the *tet*E ARG (Figure 2). The influent solids contained two ASVs of the *tet*E ARG. ASV-1 was detected in the reactors even without immigration, whilst ASV-2 did not successfully immigrate. The NCBI database returned no matches with 100% identity for sequence variant 1. The hosts of ASV-2 appeared to somewhat overlap with those of ASV-3 that was detected within the reactors. Interestingly, it was observed that ASV-2 was primarily reported to occur on plasmids in the NCBI database (NCBI Research Coordinators, 2013), whilst ASV-3 was more commonly chromosomal.

## 4. Discussion

### 4.1. Immigration impacted ARG relative abundance in the activated sludge

Droplet digital PCR revealed that the relative abundance of 11 of the 15 ARGs quantified increased with immigration. The previous analysis of the community composition data of the current reactor experiment demonstrated that the impact of immigration was better visualized using the Jaccard dissimilarity than the Bray-Curtis dissimilarity, suggesting that immigration impacts the composition of lower-abundance taxa in the mixed liquor (Gibson et al., 2023a). Consistent with this result, Procrustes analysis revealed a significant correlation between the microbial community composition and ARG profiles only when visualized with Jaccard dissimilarity. Taken together, this suggests that the changes in the ARG profiles observed is due to immigration of taxa mainly occurring at low abundances within the activated sludge.

Whilst the concentrations of the majority of ARGs increased with immigration, others such as *bla*OXA and *qac*L did not significantly change (Figure 2). The presence of these genes in similar abundance under all reactor conditions suggests that they are likely associated with the core resident populations, which were defined as taxa that occurred under all reactor conditions (i.e., independent of immigration).

### 4.2. Dynamics of ARG amplicon sequence variants between influent and activated sludge

Immigration impacted the concentration and diversity of ARGs in the activated sludge. However, it remained to be seen whether the increase in the abundance of ARGs with immigration was due to direct ARG transport or other changes associated with influent immigration. Recent studies have demonstrated ARG-ASVs to occur in samples originating from different sources (Zhang et al., 2021). Therefore, it was hypothesized that the influent wastewater, which is strongly influenced by anthropogenic activities (Guo et al., 2019), could contain different ARG-ASVs compared to the activated sludge. The detection of unique ARG-ASVs would enable a more detailed analysis of the immigration dynamics between the influent and activated sludge.

Based upon the neutral model and the assumption of ecological equivalence (i.e., equal fitness of competitors), ASVs detected in the influent would be expected to immigrate into the activated sludge (Harris et al., 2017). In both group 3 and 4, unique ASVs were observed in the influent, which could have been introduced into the reactors with immigration. However, in only one instance was ARG-ASV immigration unequivocally observed in the current study (Figure 4). Among the *bla*MOX gene, a new ASV was introduced into the reactors with immigration (Figure 4). With the introduction of this sequence ASV, the indigenous *bla*MOX sequence ASV-2, which was present without immigration, was no longer detected, suggesting either competition among the hosts of these ARG-ASVs or simply that the low concentration of the indigenous resident ASV was overwhelmed by the immigrant ASV. The *bla*MOX ASV-1 was also detected in the reactors receiving sterile influent solids, suggesting that the gene persisted in the influent solids and activated sludge reactors after autoclaving. Counter selection of ARG-ASVs between the influent and activated sludge was more frequently observed, suggesting that ARG immigration is likely not dictated solely by neutral processes and is impacted by other factors. This conclusion is supported by recent studies which have demonstrated that in WWTP samples obtained from five different countries, the resistome composition varies between the influent and activated sludge (Dai et al., 2022).

Counter selection of ARG ASVs was frequently observed between the influent and activated sludge, which suggested that direct immigration of ARGs was not occurring between the two environments. Despite this, significant increases in the relative abundances of many of these ARGs were observed in the activated sludge with immigration. Supported by the results from Procrustes analysis which showed a positive correlation between the microbial community and ARGs, this suggests that the relative abundance was instead impacted by the microbial community changes caused by immigration. This is consistent with previous studies that have identified the microbial community composition to be the main driver of AMR content in numerous environments (Munck et al., 2015; Luo et al., 2017).

The results from ARG amplicon sequencing demonstrate the complexity of ARG dynamics at the interface between the influent and activated sludge. Given the frequent observation of counter selection of ARG-ASVs, PCR based approaches alone cannot be used to accurately predict the AMR the activated sludge or infer the origin of ARGs. These data also support the contrasting results reported in the literature, which are likely influenced by the methods used and targets selected. Future studies should consider the use of amplicon sequencing approaches to enable a more accurate assessment of ARG dynamics and source tracking.

### 4.3. Genetic context of ARG amplicon sequence variants

Given the differences in the environmental conditions and microbial community between the influent and mixed liquor, counter selection of ARG-ASVs was not surprising. The successful immigration of a given ARG-ASV is likely associated with the ability of the host to adapt and establish itself under different environmental conditions, or the mobility of the gene that may be an indicator of the likelihood of transfer to hosts better adapted to the reactor environment. To further investigate these factors, ARG-ASVs were analyzed using the NCBI database and PLSDB (Galata et al., 2019).

In group 2, analysis of the *bla*TEM sequences revealed the two ARG-ASVs to have been previously reported in similar bacterial hosts and both to occur in plasmids (Supplementary Table 3). Within the influent and reactor samples *bla*TEM ASV-1 was most frequently observed. Plasmids containing this ASV have been previously detected in a diverse range of environments (Galata et al., 2019). Given that both *bla*TEM ASVs were detected among similar hosts, it could be suggested that the persistence of ASV-1 is linked to the ability of plasmids containing this ASV to move between environments.

In group 3, using multiplexed amplicon sequencing two ASVs of the *dfr*A gene were identified. The *dfr*A ASV-1 corresponded to the *dfr*A-5 gene, and ASV-2 the *dfr*A-14 variant of the gene. A 2011 review of class 1 and 2 integrons in pathogenic gram-negative bacteria identified *dfr*A-5 (ASV-1) in chicken and pig samples, as well as estuarine and carriage water. In the same study, the dfrA-14 gene was reported in pig, cattle and chicken samples, but not in aquatic settings (Stokes and Gillings, 2011). More recently, *dfr*A-14 has been observed in surface waters (Kohler et al., 2020) and WWTP effluent (Che et al., 2022). The widespread detection of both ASV-1 and ASV-2 in clinical, agricultural, and environmental settings does not support the theory of selection due to niche differences between the influent and reactors. However, both ASV-1 and 2 have been observed in similar hosts, suggesting competition may occur resulting in the loss of ASV-2 (Supplementary Table 3).

In group 4, *tet*E ASV-2 appeared most frequently in plasmids, ASV-3 was more commonly reported to be chromosomal. Previous studies have demonstrated chromosomal mutations to carry a larger fitness cost than plasmid acquired resistance (Vogwill and Maclean, 2015), however, ASV-3 was found to persist in the reactors and ASV-2 was undetected. ASV-2 has been associated with numerous plasmids found in water environments including PC1579, a conjugative plasmid recently reported to carry a novel Metallo-β-lactamase gene (Cheng et al., 2021), and plasmid pWLK-NDM, which was identified in environmental isolates carrying *bla*NDM-1 and *bla*KPC-2 resistance genes (Dang et al., 2020). Given the previous reports of ASV-2 in aquatic environments, it would be expected that ARB carrying this gene would be capable of growing within the reactor environment. However, ASV-2 remained undetected in the activated sludge suggesting again that competition for resources may be occurring.

### 4.4. Development of multiplexed amplicon sequencing

Multiplexed amplicon sequencing of ARGs is a relatively new technique requiring optimisation to ensure all primer pairs work successfully in tandem (Smith et al., 2022; Gibson et al., 2023b). Overall, eleven of the fifteen ARG targets produced data on ASV. Of the primers not successfully producing ARG-ASV information, this was often due to low levels of amplification. Considering the stringent Poisson distribution-based filters applied to the amplicon sequencing data, low count ASVs may also have been excluded during data processing. Future method developments should optimize primer design to ensure sufficient sequencing reads are obtained for all positive targets to improve resolution.

Future development of the multiplexed amplicon sequencing approach should also carefully consider which region of the ARG sequence to target. Among group 1 of concurrence profiles, low sequence diversity was found in the targeted region of the gene. The multiplexed amplicon sequence targets used herein with Illumina MiSeq protocols include only a small section of the ARG sequence (around 275 bp). Consequently, sequence diversity in other regions may have been missed. By considering regions of high genetic diversity in future design, this technique could be optimized to cover mutations of concern for example those impacting the phenotypic resistance.

### 4.5. Future application of ARG sequence diversity analysis

The monitoring of ARG ASVs at the immigration interface revealed various immigration patterns such as (i) suppression of the indigenous activated sludge ASV by the immigrant, or conversely (ii) counter selection and complete immigration failure of the influent ASV. These immigration profiles are reported for the first time here and highlight the crucial information that can be gained using our multiplex amplicon sequencing techniques.

Unique ASVs were observed among the influent and reactor samples, which highlights the potential for amplicon sequencing approaches to be utilized in the future for ARG source tracking purposes. To enable this, widespread sampling of reservoirs of antimicrobial resistance should be considered to identify ARG ASV markers present in different environments. Compared to techniques such as full metagenome sequencing, the novel amplicon sequencing approach applied here is relatively low cost and produces data that is more easily managed. In the future, such techniques could be applied for source tracking of ARG contamination in the environment and assessment of potential ARG mobility.

## Data availability statement

The datasets presented in this study can be found in online repositories. The names of the repository/repositories and accession number(s) can be found below: The European Nucleotide Archive, ERP146025.

## Author contributions

CG designed the study, conducted the digital droplet PCR, developed the multiplexed amplicon sequencing approach, analyzed the data, and prepared the manuscript. SK developed the multiplexed amplicon sequencing approach, developed the bioinformatics pipeline, processed the sequencing data, and reviewed the manuscript. NK developed the multiplexed amplicon sequencing approach and prepared samples for sequencing. DF obtained funding, supervised the research, and revised the manuscript. All authors contributed to the article and approved the submitted version.

## References

[B1] Abadii A. T. B. Rizvanov A. A. Haertlé T. Blatt N. L. (2019). World Health Organization report: Current crisis of antibiotic resistance. *Bionanoscience* 9 778–788. 10.1007/s12668-019-00658-4

[B2] Alcock B. P. Raphenya A. R. Lau T. T. Y. Tsang K. K. Bouchard M. Edalatmand A. (2020). CARD 2020: Antibiotic resistome surveillance with the comprehensive antibiotic resistance database. *Nucleic Acids Res.* 48 D517–D525. 10.1093/NAR/GKZ935 31665441 PMC7145624

[B3] Armbruster D. A. Pry T. (2008). Limit of blank, limit of detection and limit of quantitation. *Clin. Biochem. Rev.* 29:S49. PMC2556583 18852857

[B4] Bharti R. Grimm D. G. (2021). Current challenges and best-practice protocols for microbiome analysis. *Brief. Bioinform.* 22 178–193. 10.1093/BIB/BBZ155 31848574 PMC7820839

[B5] Boeije G. Corstanje R. Rottiers A. Schowanek D. (1999). Adaptation of the CAS test system and synthetic sewage for biological nutrient removal. *Chemosphere* 38 699–709. 10.1016/S0045-6535(98)00311-7 10903104

[B6] Callahan B. J. Mcmurdie P. J. Rosen M. J. Han A. W. Johnson A. J. A. Holmes S. P. (2016). DADA2: High-resolution sample inference from illumina amplicon data. *Nat. Methods* 13 581–587. 10.1038/nMeth.3869 27214047 PMC4927377

[B7] Che Y. Xia Y. Liu L. Li A. D. Yang Y. Zhang T. (2019). Mobile antibiotic resistome in wastewater treatment plants revealed by nanopore metagenomic sequencing. *Microbiome* 7:44. 10.1186/S40168-019-0663-0/FIGURES/5 PMC6429696 30898140

[B8] Che Y. Xu X. Yang Y. Bøinda K. Hanage W. Yang C. (2022). High-resolution genomic surveillance elucidates a multilayered hierarchical transfer of resistance between WWTP- and human/animal-associated bacteria. *Microbiome* 10:16. 10.1186/S40168-021-01192-W/FIGURES/6 PMC8790882 35078531

[B9] Cheng Q. Zheng Z. Ye L. Chen S. (2021). Identification of a novel metallo-β-Lactamase, VAM-1, in a foodborne *Vibrio alginolyticus* isolate from China. *Antimicrob. Agents Chemother.* 65:e0112921. 10.1128/AAC.01129-21 34424042 PMC8522725

[B10] Dai D. Brown C. Bürgmann H. Larsson D. G. J. Nambi I. Zhang T. (2022). Long-read metagenomic sequencing reveals shifts in associations of antibiotic resistance genes with mobile genetic elements from sewage to activated sludge. *Microbiome* 10:20. 10.1186/S40168-021-01216-5/FIGURES/6 PMC8801152 35093160

[B11] Dang B. Zhang H. Li Z. Ma S. Xu Z. (2020). Coexistence of the blaNDM-1-carrying plasmid pWLK-NDM and the blaKPC-2-carrying plasmid pWLK-KPC in a *Raoultella ornithinolytica* isolate. *Sci. Rep.* 10:2360. 10.1038/s41598-020-59341-4 32047243 PMC7012882

[B12] Forsberg K. J. Patel S. Gibson M. K. Lauber C. L. Knight R. Fierer N. (2014). Bacterial phylogeny structures soil resistomes across habitats. *Nat. Lett.* 509 612–616. 10.1038/nature13377 24847883 PMC4079543

[B13] Galata V. Fehlmann T. Backes C. Keller A. (2019). PLSDB: A resource of complete bacterial plasmids. *Nucleic Acids Res.* 47 D195–D202. 10.1093/NAR/GKY1050 30380090 PMC6323999

[B14] Gao P. Munir M. Xagoraraki I. (2012). Correlation of tetracycline and sulfonamide antibiotics with corresponding resistance genes and resistant bacteria in a conventional municipal wastewater treatment plant. *Sci. Total Environ.* 421–422 173–183. 10.1016/J.SCITOTENV.2012.01.061 22369865

[B15] Gibson C. Kraemer S. A. Klimova N. Vanderweyen L. Klai N. Guo B. (2023b). *Multiplexed amplicon sequencing reveals high allelic diversity of antibiotic resistance genes in Québec sewers.* bioRxiv [Preprint]. 10.1101/2023.03.06.531290

[B16] Gibson C. Jauffur S. Guo B. Frigon D. (2023a). Activated sludge microbial community assembly: The role of influent microbial community immigration. *bioRxiv* [Preprint]. 10.1101/2023.01.25.525574 PMC11337844 38995046

[B17] Guo B. Liu C. Gibson C. Frigon D. (2019). Wastewater microbial community structure and functional traits change over short timescales. *Sci. Total Environ.* 662 779–785. 10.1016/j.scitotenv.2019.01.207 30708293

[B18] Harris K. Parsons T. L. Ijaz U. Z. Lahti L. Holmes I. Quince C. (2017). Linking statistical and ecological theory: Hubbell’s unified neutral theory of biodiversity as a hierarchical Dirichlet process. *Proc. IEEE* 105 516–529. 10.1109/JPROC.2015.2428213

[B19] Jankowski P. Gan J. Le T. McKennitt M. Garcia A. Yanaç K. (2022). Metagenomic community composition and resistome analysis in a full-scale cold climate wastewater treatment plant. *Environ. Microbiomes* 17 1–20. 10.1186/S40793-022-00398-1/TABLES/2 PMC8760730 35033203

[B20] Kohler P. Tijet N. Kim H. C. Johnstone J. Edge T. Patel S. N. (2020). Dissemination of verona integron-encoded metallo-β-lactamase among clinical and environmental Enterobacteriaceae isolates in Ontario, Canada. *Sci. Rep.* 10:18580. 10.1038/s41598-020-75247-7 33122675 PMC7596063

[B21] Lapara T. M. Burch T. R. McNamara P. J. Tan D. T. Yan M. Eichmiller J. J. (2011). Tertiary-treated municipal wastewater is a significant point source of antibiotic resistance genes into Duluth-superior harbor. *Environ. Sci. Technol.* 45 9543–9549. 10.1021/ES202775R/SUPPL_FILE/ES202775R_SI_001.PDF 21981654

[B22] Le T. H. Ng C. Tran N. H. Chen H. Gin K. Y. H. (2018). Removal of antibiotic residues, antibiotic resistant bacteria and antibiotic resistance genes in municipal wastewater by membrane bioreactor systems. *Water Res.* 145 498–508. 10.1016/J.WATRES.2018.08.060 30193193

[B23] Liu S. S. Qu H. M. Yang D. Hu H. Liu W. L. Qiu Z. G. (2018). Chlorine disinfection increases both intracellular and extracellular antibiotic resistance genes in a full-scale wastewater treatment plant. *Water Res.* 136 131–136. 10.1016/J.WATRES.2018.02.036 29501757

[B24] Luo G. Li B. Li L.-G. Zhang T. Angelidaki I. (2017). Antibiotic resistance genes and correlations with microbial community and metal resistance genes in full-scale biogas reactors as revealed by metagenomic analysis. *Environ. Sci. Technol.* 51 4069–4080. 10.1021/acs.est.6b05100 28272884

[B25] Munck C. Albertsen M. Telke A. Ellabaan M. Nielsen P. H. Sommer M. O. A. (2015). Limited dissemination of the wastewater treatment plant core resistome. *Nat. Commun.* 6:8452. 10.1038/ncomms9452 26419330 PMC4598724

[B26] Munir M. Wong K. Xagoraraki I. (2011). Release of antibiotic resistant bacteria and genes in the effluent and biosolids of five wastewater utilities in Michigan. *Water Res.* 45 681–693. 10.1016/J.WATRES.2010.08.033 20850863

[B27] NCBI Research Coordinators (2013). Database resources of the National Center for Biotechnology Information. *Nucleic Acids Res.* 41 D8–D20. 10.1093/NAR/GKS1189 23193264 PMC3531099

[B28] O’Neill J. (2014). *Review on antimicrobial resistance. Antimicrobial Resistance: Tackling a crisis for the health and wealth of nations.* London: Wellcome Trust.

[B29] Oksanen J. Simpson G. L. Blanchet F. G. Kindt R. Legendre P. Minchin P. R. (2022). *Vegan: Community ecology package.*

[B30] Pang Y. Huang J. Xi J. Hu H. Zhu Y. (2016). Effect of ultraviolet irradiation and chlorination on ampicillin-resistant *Escherichia coli* and its ampicillin resistance gene. *Front. Environ. Sci. Eng.* 10:522–530. 10.1007/s11783-015-0779-9

[B31] Qin K. Wei L. Li J. Lai B. Zhu F. Yu H. (2020). A review of ARGs in WWTPs: Sources, stressors and elimination. *Chin. Chem. Lett.* 31 2603–2613. 10.1016/J.CCLET.2020.04.057

[B32] Quintela-Baluja M. Abouelnaga M. Romalde J. Su J. Q. Yu Y. Gomez-Lopez M. (2019). Spatial ecology of a wastewater network defines the antibiotic resistance genes in downstream receiving waters. *Water Res.* 162 347–357. 10.1016/J.WATRES.2019.06.075 31295654 PMC6650630

[B33] Reichert G. Hilgert S. Alexander J. Rodrigues de Azevedo J. C. Morck T. Fuchs S. (2021). Determination of antibiotic resistance genes in a WWTP-impacted river in surface water, sediment, and biofilm: Influence of seasonality and water quality. *Sci. Total Environ.* 768:144526. 10.1016/J.SCITOTENV.2020.144526 33450684

[B34] Rizzo L. Manaia C. Merlin C. Schwartz T. Dagot C. Ploy M. C. (2013). Urban wastewater treatment plants as hotspots for antibiotic resistant bacteria and genes spread into the environment: A review. *Sci. Total Environ.* 447 345–360. 10.1016/j.scitotenv.2013.01.032 23396083

[B35] Sánchez-Osuna M. Cortés P. Llagostera M. Barbé J. Erill I. (2020). Exploration into the origins and mobilization of di-hydrofolate reductase genes and the emergence of clinical resistance to trimethoprim. *Microb. Genomics* 6:mgen000440. 10.1099/mgen.0.000440 32969787 PMC7725336

[B36] Smith S. D. Choi J. Ricker N. Yang F. Hinsa-Leasure S. Soupir M. L. (2022). Diversity of antibiotic resistance genes and transfer elements-quantitative monitoring (DARTE-QM): A method for detection of antimicrobial resistance in environmental samples. *Commun. Biol.* 5:216. 10.1038/s42003-022-03155-9 35301418 PMC8931014

[B37] Staley C. Kaiser T. Vaughn B. P. Graiziger C. Hamilton M. J. Kabage A. J. (2019). Durable long-term bacterial engraftment following encapsulated fecal microbiota transplantation to treat *Clostridium difficile* infection. *mBio* 10 e01586-19. 10.1128/mbio.01586-19 31337728 PMC6650559

[B38] Stokes H. W. Gillings M. R. (2011). Gene flow, mobile genetic elements and the recruitment of antibiotic resistance genes into gram-negative pathogens. *FEMS Microbiol. Rev.* 35 790–819. 10.1111/j.1574-6976.2011.00273.x 21517914

[B39] Sun S. Geng J. Li B. Ma L. Sun X. Meng F. (2021). Temporal variations of antibiotic resistance genes in influents and effluents of a WWTP in cold regions. *J. Clean. Prod.* 328:129632. 10.1016/J.JCLEPRO.2021.129632

[B40] United Nations Environment Programme (2022). *Environmental dimensions of antimicrobial resistance: Summary for policymakers.* Nairobi: United Nations Environment Programme.

[B41] Vogwill T. Maclean R. C. (2015). The genetic basis of the fitness costs of antimicrobial resistance: A meta-analysis approach. *Evol. Appl.* 8 284–295. 10.1111/eva.12202 25861386 PMC4380922

[B42] Von Wintersdorff C. J. H. Penders J. Van Niekerk J. M. Mills N. D. Majumder S. Van Alphen L. B. (2016). Dissemination of antimicrobial resistance in microbial ecosystems through horizontal gene transfer. *Front. Microbiol.* 7:173. 10.3389/FMICB.2016.00173/BIBTEX PMC4759269 26925045

[B43] Wu D. Huang X.-H. Sun J.-Z. Graham D. W. Xie B. (2017). Antibiotic resistance genes and associated microbial community conditions in aging landfill systems. *Environ. Sci. Technol.* 51 12859–12867. 10.1021/acs.est.7b03797 28990771

[B44] Xia S. Jia R. Feng F. Xie K. Li H. Jing D. (2012). Effect of solids retention time on antibiotics removal performance and microbial communities in an A/O-MBR process. *Bioresour. Technol.* 106 36–43. 10.1016/J.BIORTECH.2011.11.112 22197076

[B45] Zaheer R. Noyes N. Ortega Polo R. Cook S. R. Marinier E. Van Domselaar G. (2018). Impact of sequencing depth on the characterization of the microbiome and resistome. *Sci. Rep.* 8:5890. 10.1038/s41598-018-24280-8 29651035 PMC5897366

[B46] Zhang A. N. Gaston J. M. Dai C. L. Zhao S. Poyet M. Groussin M. (2021). An omics-based framework for assessing the health risk of antimicrobial resistance genes. *Nat. Commun.* 12:4765. 10.1038/s41467-021-25096-3 34362925 PMC8346589

[B47] Zhang M. Chen S. Yu X. Vikesland P. Pruden A. (2019). Degradation of extracellular genomic, plasmid DNA and specific antibiotic resistance genes by chlorination. *Front. Environ. Sci. Eng.* 13:38. 10.1007/s11783-019-1124-5

[B48] Zhou Z.-C. Zheng J. Wei Y.-Y. Chen T. Dahlgren R. A. Shang X. (2017). Antibiotic resistance genes in an urban river as impacted by bacterial community and physicochemical parameters. *Environ. Sci. Pollut. Res.* 24 23753–23762. 10.1007/s11356-017-0032-0 28864929

[B49] Zhu Y. Wang Y. Zhou S. Jiang X. Ma X. Liu C. (2018). Robust performance of a membrane bioreactor for removing antibiotic resistance genes exposed to antibiotics: Role of membrane foulants. *Water Res.* 130 139–150. 10.1016/J.WATRES.2017.11.067 29216481

