# Supplementary material for: Antibiotic resistance gene sequencing is necessary to reveal the complex dynamics of immigration from sewers to activated sludge
Source: Front Microbiol. 2023 Apr 26;14:1155956. doi: 10.3389/fmicb.2023.1155956 (PMC10204801; doi:10.3389/fmicb.2023.1155956)
Supplement: Supplementary file 1 [file Data_Sheet_1.docx]

Antibiotic Resistance Gene Sequencing is Necessary to Reveal the Complex Dynamics of Immigration from Sewers to Activated Sludge

Claire Gibson^1^, Susanne A. Kraemer^2^, Natalia Klimova^1^, Bing Guo^3^ & Dominic Frigon^1*^

^1^McGill University, Department of Civil Engineering and Applied Mechanics, Montreal, Quebec, Canada

^2^Environment and Climate Change Canada, Montreal, Quebec, Canada

^3^Centre for Environmental Health and Engineering, Department of Civil and Environmental Engineering, University of Surrey, United Kingdom

*** Correspondence:**Dominic Frigon

Dominic.Frigon@mcgill.ca


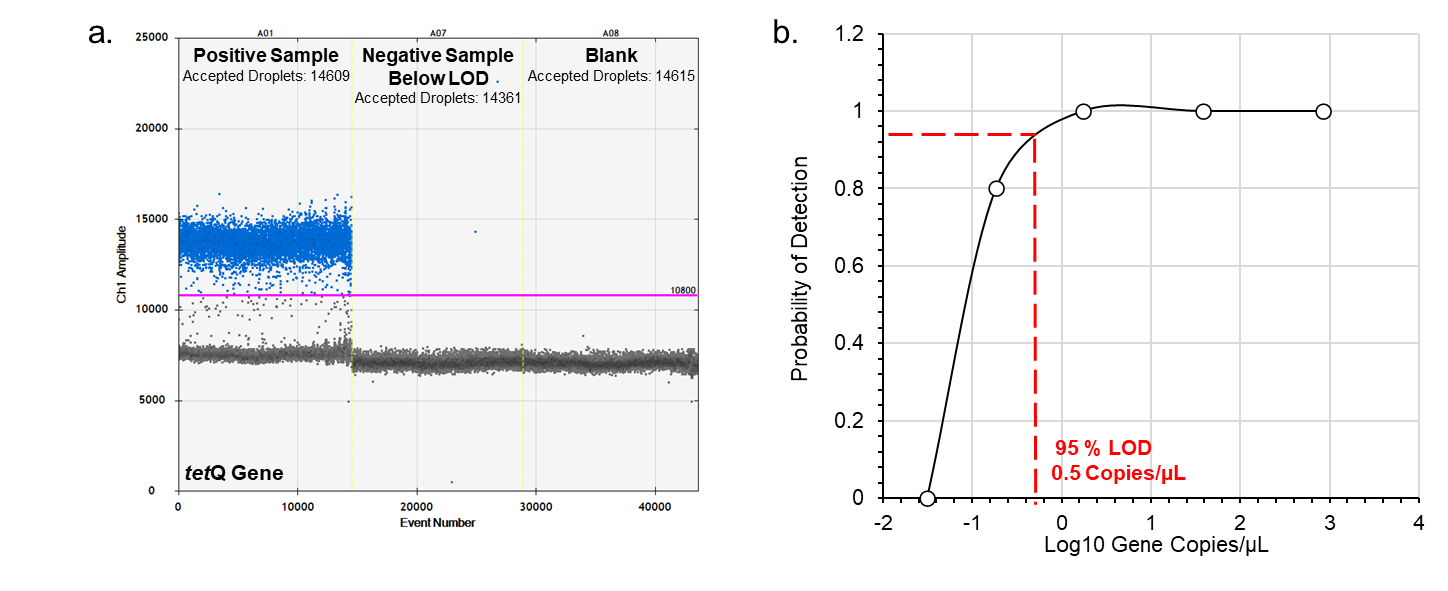


Figure S1- Digital droplet PCR for ARG detection a) Example of QuantaSoft™ droplet fluorescence analysis demonstrating a positive result, a result below the limit of detection and a no template control (blank) b) Determining the 95 % limit of detection for the tetQ ARG. Each sample was analysed 6 times in 2 different PCR runs (3 samples per run).

| **Table S1**- Primers used for droplet digital PCR | | | |  |
| --- | --- | --- | --- | --- |
| Target | Forward Sequence (5’-3’) | Reverse Sequence (5’-3’) | Annealing Temp. (°C) | Resistance Drug Class^1^ |
| *bla*MOX | ACCAGCTCGGCGGATCTG | GAGCCGGTCTTGTTGAAGAGC | 65 | Beta-Lactam |
| *bla*OXA | AGGCACGATAGTTGTGGCAGAC | GTAGAATTCCGCATTGCTGATCGC | 65 | Beta-Lactam |
| *bla*TEM | GAACCGGAGCTGAATGAAGCC | CGGGAGGGCTTACCATCTGG | 65 | Beta-Lactam |
| *dfr*A | ACATACCCTGGTCCGCGAAAG | CGCCACCAGACACTATAACGTGA | 65 | Diaminopyrimidines |
| *ere*B | CAGCTCATCGATCACCTCATGAAACCG | CACGTACGGAAGTATCTCCCTCAA | 64 | Macrolide |
| *mar*R | CACAGTTTAAGGTGCTCTGCTCTATCC | GCAAATACTCAAGTGTTGCCACTTCG | 63 | Multi-drug |
| *mph*E | AAGTGAGCAATTGGAAACCCGCTA | AGGCCGCTGCTCTTTCTAAAGTC | 65 | Macrolide |
| *msr*D | GGCAAGCTAGGTGTTGAGCAATTAG | TCCTTCACGGTCTAAATGGCTCGTA | 65 | Multi-drug |
| *qac*L | GTTGCAATCTTTGGCGAGGTCA | CGCTGACCTTGGATAGCAGGTTTAGAAC | 63 | Multi-drug |
| *qnr*B | CGACCTGAGCGGCACTGAATTTA | GCTCGCCAGTCGAAAGTCGAA | 65 | Fluoroquinolones |
| *qnr*S | ATGCCAGCTTGCGATGGCAAA | GTGGCATAAATTAGCACCCTGTAGGC | 65 | Fluoroquinolones |
| *rob*A | TCAAATGCGCGTGCAGTTCTGG | GTAGCGCTCAATATCCTGACCTTTAC | 63 | Multi-drug |
| *tet*E | TGATTGCTGGACCAGTCATTGG | CCATACGAAGCGCTCTTCTCC | 64 | Tetracycline |
| *tet*O | GCAGGGACAGAACTATTAGAGCCATATC | GCTAACTTGTGGAACATATGCCGAAC | 64 | Tetracycline |
| *tet*Q | TGGATTGAAGACCCGTCTTTGTCC | AGCAGGTGTACTTACCGGGCTATA | 65 | Tetracycline |
|  | | | | |

| **Table S2-** Log change in ARG between the influent and mixed liquor | | | | | | | |
| --- | --- | --- | --- | --- | --- | --- | --- |
|  | Log Copies/16S rRNA | |  | Log Copies/L | |  | Log change |
| Gene | Influent | Reactors |  | Influent | Reactors |  |  |
| *bla*MOX | -2.14 + 0.31 | -3.38 + 0.13 |  | 7.43 | 6.91 |  | -0.52 |
| *bla*TEM | -2.20 + 0.06 | -3.87 + 0.13 |  | 7.26 | 6.41 |  | -0.85 |
| *bla*OXA | -1.68 + 0.41 | -2.03 + 0.29 |  | 7.89 | 8.31 |  | 0.42 |
| *qnr*S | -2.18 + 0.32 | -3.81 + 0.16 |  | 7.37 | 6.48 |  | -0.89 |
| *qnr*B | -1.86 + 0.15 | -3.67 +0.18 |  | 7.62 | 6.63 |  | -0.99 |
| *dfr*A | -2.11 + 0.10 | -3.79 + 0.16 |  | 7.35 | 6.50 |  | -0.85 |
| *mph*E | -1.34 + 0.15 | -2.39 + 0.15 |  | 8.16 | 7.90 |  | -0.26 |
| *ere*A | -2.88 + 0.01 | -4.09 + 0.05 |  | 6.58 | 6.18 |  | -0.40 |
| *tet*O | -2.88 + 0.05 | -3.86 + 0.01 |  | 6.58 | 6.41 |  | -0.17 |
| *tet*Q | -2.09 + 0.07 | -3.74 + 0.11 |  | 7.38 | 6.53 |  | -0.84 |
| *tet*E | -2.89 + 0.41 | -3.54 + 0.13 |  | 6.71 | 6.74 |  | 0.04 |
| *rob*A | -2.99 + 0.07 | -4.95 + 0.13 |  | 6.47 | 5.33 |  | -1.14 |
| *msr*D | -2.57 + 0.12 | -3.69 + 0.07 |  | 6.89 | 6.58 |  | -0.31 |
| *qac*L | -0.43 + 0.12 | -2.32 + 0.07 |  | 9.06 | 7.92 |  | -1.11 |
| *mar*R | -3.06 + 0.27 | -4.75 + 0.10 |  | 6.44 | 5.52 |  | -0.92 |
| Log copies calculated by multiplying copies 16S rRNA gene/ng-DNA, Yield DNA/VSS, reactor VSS and relative ARG concentration. These values are available in Table S4 of Gibson et al., 2023. | | | | | | | |

| **Table S3**- Sequence Variants Obtained from Targeted Amplicon Sequencing (Figure 4 & 5) | | | | | |
| --- | --- | --- | --- | --- | --- |
| Gene | Allele Sequence | | NCBI BLAST/CARD Result | | |
| *bla*MOX  ASV 1  ASV 2 | >consensus_1  CTGAAGTTTGTCGGCGCCAACATGACAGGCACCGGGGACGAGGCGATGCAGCAGGCGATTGCCCTGACCCACAAGGGGGTTTACTCGGTGGGTGCCATGACTCAGGGGCTCGGCTGGGAGAGTTACGCCTATCCCGTGACCGAAGAGACCTTGCTTGCAGGCAACTCGGGCAAGGTGATCCTCGAGGCCAACCCGACGGCGCCCGCCTCCAACGAGACGGGTAGCCAGGT | | *Aeromonas media*  Uncultured bacterium |  | |
|  | >consensus_2  CTGCGCTTTGTGAAGGCCAACATCAGCGGGGTGGATAATGCGGCCATGCAGCAGGCCATCGATCTGACTCACCAGGGCCAGTATGCGGTGGGGGAGATGACCCAGGGACTGGGCTGGGAGCGTTACCCCTATCCCGTCAGCGAGCAGACGCTGCTGGCGGGCAACTCCCCGGCGATGATTTACAATGCCAACCCGGCGGCGCCCGCGCCCGCTGCGGCAGGGCACCCTGT | | No 100% identity found  Highest percentage identity reported: 99.13 % Query Cover: 100 % |  | |
| *tet*E  ASV 1  ASV 2 | >consensus_1  TGGTTTCCGATCTTGATTGCTGGACCAGTCATTGGTGGTTTTGCAGGTCAACTTTCGGTACAGGCACCGTTTATGTTCGCTGCTGCCATTAACGGGCTGGCATTTCTGGTCTCCCTATTCATTTTACATGAGACCCATAATGCTAATCAGGTTAGTGACGAGATAAAGAATGAAACAATCAATGAAACCACATCCTCCATACGCGAGATGATCTCCCCATTATCGGGATTGCTAGTTGTCTTTTTCATCATTCAATTGATTGGCCAAATCCCTGCAACATTATGGGTTTTATTC | | No 100% identity found  Highest percentage identity reported: 100 % Query Cover: 95 % |  | |
|  | >consensus_2  TGGTTTTGCAGGTCAACTTTCGGTACAGGCACCGTTTATGTTCGCTGCTGCTATTAACGGGCTGGCATTTCTGGTCTCCCTATTCATTTTACATGAGACCCATAATGCTAATCAGGTTAGTGACGAGATAAAGAATGAAACAATCAATGAAACCACATCCTCCATACGCGAGATGATCTCCCCATTATCGGGATTGCTAGTTGTCTTTTTCATCATTCAATTGATTGGCCAAATCCCTGCAACATTATGGGTTTTATTC | | *Aeromonas caviae*  *Aeromonas hydrophilia*  *Aeromonas media*  *Aeromonas salmonicida*  *Aeromonas veronii*  *Raoultella ornithinolytica*  *Vibrio alginolyticus*  *Vibrio parahaemolyticus* |  | |
| Gene | Allele Sequence | | NCBI BLAST/CARD Result | | |
| *tet*E (continued)  ASV 3 | >consensus_3  TGGTTTTGCCGGTCAACTTTCGGTACAGGCACCGTTTATGTTCGCTGCTGCTATTAACGGGCTGGCATTTCTGGTCTCCCTATTCATTTTACATGAGACCCATAATGCTAATCAGGTTAGTGACGAGTTAAAGAATGAAACAATCAATGAAACCACATCCTCCATACGCGAGATGATCTCCCCATTATCGGGATTGTTAGTTGTCTTTTTCATCATTCAATTGATTGGCCAAATCCCCGCAACATTATGGGTTTTATTC | | *Aeromonas caviae*  *Aeromonas media*  *Aeromonas veronii*  *Aeromonas sp.*  *Enterobacter cloacae*  *Yersinia ruckeri*  *Escherichia coli*  *Aeromonas hydrophila*  *Aeromonas dhakensis* | | |
| *bla*TEM  ASV 1 | >consensus_1  ATACCAAACGACGAGCGTGACACCACGATGCCTGCAGCAATGGCAACAACGTTGCGCAAACTATTAACTGGCGAACTACTTACTCTAGCTTCCCGGCAACAATTAATAGACTGGATGGAGGCGGATAAAGTTGCAGGACCACTTCTGCGCTCGGCCCTTCCGGCTGGCTGGTTTATTGCTGATAAATCTGGAGCCGGTGAGCGTGGGTCTCGCGGTATCATTGCAGCACTGGGG | *Acinetobacter baumannii*  *Acinetobacter johnsonii*  *Acinetobacter towneri*  *Aeromonas hydrophila*  *Aeromonas veronii*  *Bacillus subtilis*  *Bacteroides fragilis*  *Chlamydia trachomatis*  *Citrobacter amalonaticus*  *Citrobacter freundii*  *Citrobacter koseri*  *Citrobacter portucalensis*  *Citrobacter werkmanii*  *Citrobacter youngae*  *Clostridioides difficile*  *Cronobacter sakazakii*  *Enterobacter asburiae*  *Enterobacter chengduensis*  *Enterobacter cloacae*  *Enterobacter hormaechei*  *Enterobacter kobei*  *Enterobacter roggenkampii*  *Enterobacter spp.*  *Enterobacteriaceae*  *Enterococcus faecium*  *Escherichia albertii*  *Escherichia coli*  *Escherichia fergusonii*  *Escherichia marmotae*  *Haemophilus influenzae* | | | \| *Haemophilus parainfluenzae* \| \| --- \| \| *Kingella kingaegi* \| \| *Klebsiella aerogenes* \| \| *Klebsiella huaxiensis* \| \| *Klebsiella michiganensis* \| \| *Klebsiella oxytoca* \| \| *Klebsiella pneumoniae* \| \| *Klebsiella quasipneumoniae* \| \| *Leclercia adecarboxylata* \| \| *Morganella morganii* \| \| *Mycobacterium tuberculosis* \| \| *Neisseria gonorrhoeae* \| \| *Pasteurella multocida* \| \| *Proteus mirabilis* \| \| *Proteus vulgaris* \| \| *Providencia rettgeri* \| \| *Providencia stuartii* \| \| *Pseudomonas aeruginosa* \| \| *Pseudomonas putida* \| \| *Raoultella planticola* \| \| *Salmonella enterica* \| \| *Serratia marcescens* \| \| *Shigella boydii* \| \| *Shigella dysenteriae* \| \| *Shigella flexneri* \| \| *Shigella sonnei* \| \| *Staphylococcus aureus* \| \| *Streptococcus suis* \| \| *Vibrio cholerae* \| \| *Vibrio parahaemolyticus* \| |
| Gene | Allele Sequence | NCBI BLAST/CARD Result | | | |
| *bla*TEM (continued)  ASV 2 | >consensus_2  ATACCAAACGACGAGCGTGACACCACGATGCCTGTAGCAATGGCAACAACGTTGCGCAAACTATTAACTGGCGAACTACTTACTCTAGCTTCCCGGCAACAATTAATAGACTGGATGGAGGCGGATAAAGTTGCAGGACCACTTCTGCGCTCGGCCCTTCCGGCTGGCTGGTTTATTGCTGATAAATCTGGAGCCGGTGAGCGTGGGTCTCGCGGTATCATTGCAGCACTGGGG | | \| *Acinetobacter baumannii* \| \| --- \| \| *Acinetobacter haemolyticus* \| \| *Bacillus cereus* \| \| *Bacillus subtilis* \| \| *Bacillus velezensis* \| \| *Bifidobacterium longum* \| \| *Burkholderia cepacia* \| \| *Burkholderia lata* \| \| *Chlamydia trachomatis* \| \| *Clostridioides difficile* \| \| *Clostridium botulinum* \| \| *Cronobacter sakazakii* \| \| *Enterobacter cloaceae* \| \| *Enterococcus faecium* \| \| *Escherichia coli* \| \| *Faecalibacterium prausnitzii* \| \| *Helicobacter pylori* \| \| *Klebsiella michiganensis* \| \| *Klebsiella pneumoniae* \| \| *Klebsiella quasipneumoniae* \| \|  \| \|  \| \|  \| \|  \| \|  \| \|  \| \|  \| \|  \| \|  \| \|  \| \|  \| \|  \| \|  \| \|  \| \|  \| \|  \| \|  \| | | \| *Leclercia adecarboxylata* \| \| --- \| \| *Legionella pneumophila* \| \| *Mycobacterium tuberculosis* \| \| *Mycoplasma mycoides* \| \| *Neisseria meningitidis* \| \| *Propionibacterium freudenreichii* \| \| *Pseudomonas aeruginosa* \| \| *Rhizobium leguminosarum* \| \| *Ruthenibacterium lactatiformans* \| \| *Salmonella enterica* \| \| *Staphylococcus aureus* \| \| *Staphylococcus hominis* \| \| *Staphylococcus saprophyticus* \| \| *Streptococcus agalactiae* \| \| *Streptococcus lutetiensis* \| \| *Streptococcus pneumoniae* \| \| *Vibrio parahaemolyticus* \| |
| *dfr*A  ASV 1  ASV 2 | >consensus_1  GGAGAGCAGCTACTCTTTAAAGCCTTGACGTACAACCAGTGGCTTTTGGTGGGCCGCAAGACGTTCGAATCTATGGGAGCACTCCCTAATAGGAAATACGCGGTCGTTACTCGCTCAGCCTGGACGGCCGATAATGACAACGTAATAGTATTCCCGTCGATCGAAGAGGCCATGTACGGGCTGGCTGAACTCACCGA | | \| *Acinetobacter baumannii* \| \| --- \| \| *Aeromonas caviae* \| \| *Citrobacter koseri* \| \| *Comamonas testosteroni* \| \| *Enterobacter hormaechei* \| \| *Enterobacter roggenkampii* \| \| *Escherichia albertii* \| \| *Escherichia coli* \| \| *Escherichia fergusonii* \| \| *Klebsiella michiganensis* \| \| *Klebsiella pneumoniae* \| \| *Klebsiella quasipneumoniae* \| | | \| *Proteus mirabilis* \| \| --- \| \| *Proteus vulgaris* \| \| *Vibrio cholerae* \| \| *Pseudomonas aeruginosa* \| \| *Salmonella enterica* \| \| *Serratia marcescens* \| \| *Shigella boydii* \| \| *Shigella flexneri* \| \| *Shigella sonnei* \| |
|  | >consensus_2  GGGGAGCAGCTACTTTTTAAAGCATTGACCTACAATCAGTGGCTTCTGGTGGGTCGCAAGACGTTTGAATCTATGGGCGCACTCCCCAATAGGAAATACGCGGTCGTTACCCGCTCAGGTTGGACATCAAATGATGACAATGTAGTTGTATTTCAGTCAATCGAAGAGGCCATGGACAGGCTAGCTGAATTCACCGG | | \| *Aeromonas hydrophila* \| \| --- \| \| *Aeromonas veronii* \| \| *Citrobacter amalonaticus* \| \| *Citrobacter freundii* \| \| *Citrobacter portucalensis* \| \| *Citrobacter werkmanii* \| \| *Citrobacter youngae* \| \| *Enterobacter asburiae* \| \| *Enterobacter cloacae* \| \| *Enterobacter hormaechei* \| \| *Enterobacter kobei* \| \| *Enterobacter roggenkampii* \| \| *Escherichia albertii* \| \| *Escherichia coli* \| \| *Escherichia fergusonii* \| \| *Klebsiella michiganensis* \| \| *Klebsiella grimontii* \| \| *Klebsiella pneumoniae* \| \| *Klebsiella quasipneumoniae* \| \|  \| | | \| *Morganella morganii* \| \| --- \| \| *Proteus mirabilis* \| \| *Proteus vulgaris* \| \| *Providencia rettgeri* \| \| *Providencia stuartii* \| \| *Pseudomonas aeruginosa* \| \| *Raoultella planticola* \| \| *Salmonella enterica* \| \| *Serratia marcescens* \| \| *Shewanella putrefaciens* \| \| *Shigella boydii* \| \| *Shigella dysenteriae* \| \| *Shigella flexneri* \| \| *Shigella sonnei* \| \| *Vibrio cholerae* \| |
| Gene | Allele Sequence | | NCBI BLAST/CARD Result | | |
| *bla*OXA  ASV 1  ASV 2  ASV 3  ASV 4 | >consensus_1  GAACGCCAAGCGGATCGTGCCATGTTGGTTTTTGATCCTGTGCGATCGAAGAAACGCTACTCGCCTGCATCGACATTCAAGATACCTCATACACTTTTTGCACTTGATGCAGGCGCTGTTCGTGATGAGTTCCAGATTTTTCGATGGGACGGCGTTAACAGGGGCTTTGCAGGCCACAATCAAGACCAAGATTTGCGATCAGCAATGCGGAATTCTACAGATCGGAAGAGCACACGTCT | | \| *Acinetobacter baumannii* \| \| --- \| \| *Aeromonas caviae* \| \| *Alcaligenes faecalis* \| \| *Burkholderia cenocepacia* \| \| *Citrobacter freundii* \| \| *Citrobacter koseri* \| \| *Citrobacter portucalensis* \| \| *Citrobacter werkmanii* \| \| *Enterobacter asburiae* \| \| *Enterobacter cloacae* \| \| *Enterobacter hormaechei* \| \| *Enterobacter kobei* \| \| *Enterobacter roggenkampii* \| \| *Escherichia coli* \| \| *Klebsiella michiganensis* \| \| *Klebsiella oxytoca* \| \|  \| \|  \| \|  \| \|  \| | | \| *Klebsiella pneumoniae* \| \| --- \| \| *Klebsiella quasipneumoniae* \| \| *Pasteurella multocida* \| \| *Proteus mirabilis* \| \| *Providencia rettgeri* \| \| *Providencia stuartii* \| \| *Pseudomonas aeruginosa* \| \| *Pseudomonas monteilii* \| \| *Pseudomonas putida* \| \| *Pseudomonas stutzeri* \| \| *Salmonella enterica* \| \| *Serratia marcescens* \| \| *Shigella sonnei* \| \| *Stenotrophomonas maltophilia* \| \| *Vibrio cholerae* \| |
|  | >consensus_2  GAACGCCAAGCGGATCGTGCCATGTTGGTTTTTGATCCTGTGCGATCGAAGAAACGCTACTCGCCTGCATCGACATTCAAGATACCTCATACACTTTTTGCACTTGATGCAGGCGCTGTTCGTGATGAGTTCCAGATTTTTCGATGGGACGGCGTTAACAGGGGCTTTGCAGGCCACAATCAAGACCAAGATTTGCGACCAGCAATGCGGAATTCTACAGATCGGAAGAGCACAC | | No 100% identity found  Highest percentage identity reported: 99.54 % Query Cover: 92 % | |  |
|  | >consensus_3  GAACGCCAAGCGGATCGTGCCATGTTGGTTTTTGATCCTGTGCGATCGAAGAAACGCTACTCGCCTGCATCGACATTCAAGATACCTCATACACTTTTTGCACTTGATGCAGGCGCTGTTCGTGATGAGTTCCAGATTTTTCGATGGGACGGCGTTAACAGGGGCTTTGCAGGCCACAATCAAGACCAAGATTTGCGATCAGCAATGCGGAATTTAC | | No 100% identity found  Highest percentage identity reported: 99.54 % Query Cover: 100 % | |  |
|  | >consensus_4  GAACGCCAAGCGGATCGTGCCATGTTGGTTTTTGATCCTGTGCGATCGAAGAAACGCTACTCGCCTGCATCGACATTCAAGATACCTCATACACTTTTTGCACTTGATGCAGGCGCTGTTCGTGATGAGTTCCAGATTTTTCGATGGGACGGCGTTAACAGGGGCTTTGCAGGCCACAATCAAGACCAAGATTTGCGATCAGCGAT | | *Pseudomonas aeruginosa*  *Pseudomonas putida*  *Stenotrophomonas maltophilia* |  | |
| Gene | Allele Sequence | | NCBI BLAST/CARD Result | | |
| *bla*OXA (continued)  ASV 5 | >consensus_5  GAACGCCAAGCGGATCGTGCCATGTTGGTTTTTGATCCTGTGCGATCGAAGAAACGCTACTCGCCTGCATCGACATTCAAGATACCTCATACACTTTTTGCACTTGATGCAGGCGCTGTTCGTGATGAGTTCCAGATTTTTCGATGGGACGGCGTTAACAGGGGCTTTGCAGGCCACAATCAAGACCAAGATTTT | | \| *Acinetobacter baumannii* \| \| --- \| \| *Aeromonas caviae* \| \| *Alcaligenes faecalis* \| \| *Burkholderia cenocepacia* \| \| *Citrobacter freundii*  *Citrobacter koseri*  *Citrobacter portucalensis*  *Citrobacter werkmanii* \| \| *Enterobacter asburiae* \| \| *Enterobacter cloacae* \| \| *Enterobacter hormaechei* \| \| *Enterobacter kobei* \| \| *Enterobacter roggenkampii* \| \| *Escherichia coli* \| \| *Klebsiella michiganensis* \| \| *Klebsiella oxytoca* \| | | \|  \| \| --- \| \| *Klebsiella pneumoniae* \| \| *Klebsiella quasipneumoniae* \| \| *Pasteurella multocida* \| \| *Proteus mirabilis* \| \| *Providencia rettgeri* \| \| *Providencia stuartii* \| \| *Pseudomonas aeruginosa* \| \| *Pseudomonas monteilii* \| \| *Pseudomonas putida* \| \| *Salmonella enterica* \| \| *Serratia marcescens* \| \| *Shigella sonnei* \| \| *Stenotrophomonas maltophilia* \| \| *Vibrio cholerae* \| |
| *qac*L  ASV 1  ASV 2 | >consensus_1  CACGACGCTCTTCCGATCTCTGTTTCAATCTTTGGCGCGGTCATCGCAACTTCCGCACTGAAGTCTAGCCATGGATTCACTAGGTTAGTTCCTTCCGTTGTAGTTGTGGCTGGCTACGGGCTTGCGTTCTATTTCTTGTCTCTCGCGCTCAAGTCCATTCCGGTCGGTATTGCTTACGCTGTATGGGCTGGGCTTGGCATCGTGCTTGTGGCAGCTATTGCTTGGATTTTCCATGGCCAAAAACTAGACTTCTGGGCGTTCATTGGCATGGGACTTAT | | No 100% identity found  Highest percentage identity reported: 99.61 % Query Cover: 93 % | |  |
|  | >consensus_2  TCGCAACTTCCGCACTGAAGTCTAGCCATGGATTCACTAGGTTAGTTCCTTCCGTTGTAGTTGTGGCTGGCTACGGGCTTGCGTTCTATTTCTTGTCTCTCGCGGTCAAGTCCATTCCGGTCGGTATTGCTTACGCTGTATGGGCTGGGCTTGGCATCGTGCTTGTGGCAGCTATTGCTTGGATTTTCCATGGCCAAAAACTAGACTTCTGGGCGTTCATTGGCATGGGACTTAT | | Uncultured prokaryote | |  |
| Gene | Allele Sequence | | NCBI BLAST/CARD Result | | |
| *mar*R  ASV 1 | >consensus_8  GCTGTGCGGGCTGCATTACCCCGGTTGAACTGAAAAAAGTGTTGTCTGTCGATCTCGGCGCCTTAACGCGCATGCTCGAGCGTCTGGTCTGCAAAGGCTGGATTGACAGACTGCCTAACCCACATGACAAACGCGGTGTGCTGGTGAAACTCACCGAACACGGCGCGGCAATTTGTGAGCAATGTCATCAATTAGTAGGACAAGACCTGCACCAGGAATTAACAAAAAACTTAACGGCGGA | | *Citrobacter*  *Citrobacter sp.*  *Citrobacter amalonaticus* | |  |
| *tet*Q  ASV 1 | >consensus_1  CTTTTCCATAAACTCATATAGTGATGAATTGGAAATCTCGTTATATGGTTTGACCCAAAAGGAAATCATACAGACATTGCTGGAAGAACGATTTTCCGTAAAGGTCCATTTTGATGAGATCAAGACTATCTACAAAGAACGACCTATAAAAAAGGTCAATAAGATTATTCAGATCGAAGTACCACCCAACCCTTACTGGGCCACAATAGGGCTGACTCTTGAACCCTTACCGTTAGGGGCAGGGTTGCAAATCGAAAGTGACATCTCCTATGGTTATCTGAACCATTCTTTTCAAAATGCCGTTTTTGAAGGGATTCGTATGTCTTGCCAATCTGGTTTACATGGATGGGAAGTGACAGATCTGAAAGTAACTTTTACTCAAGCCGAGTAT | | *Alistipes communis*  *Alistipes onderdonkii*  *Bacteroides caccae*  *Bacteroides caecimuris*  *Bacteroides cellulosilyticus*  *Bacteroides dorei*  *Bacteroides eggerthii*  *Bacteroides fragilis*  *Bacteroides ovatus*  *Bacteroides salyersiae*  *Bacteroides sp.*  *Bacteroides thetaiotaomicron*  *Bacteroides uniformis*  *Bactroides xylanisolvens*  *Bacteroides zhangwenhongii*  *Butyricimonas faecalis*  *Coprobacter secundus*  *Odoribacteraceae bacterium* | | *Parabacteroides distasonis*  *Parabacteroides goldsteinii*  *Parabacteroides johnsonii*  *Parabacteroides merdae*  *Parabacteroides sp.*  *Paraprevotella xylaniphila*  *Phocaeicola dorei*  *Phocaeicola vulgatus*  *Prevotella buccalis*  *Prevotella intermedia*  *Prevotella melaninogenica*  *Prevotella ruminocola*  *Prevotella sp.*  *Pseudoprevotella muciniphila*  *Riemerella anatipestifer*  *Sodaliphilus pleomorphus*  *Uncultured bacterium* |
| *mph*E  ASV 1 | >consensus_2  CAGAAAATGGTTGGATAATGATGTTCTATGGGCAGATTTCACCCAATTTATACATGGCGATTTATATGCTGGGCATGTACTAGCTTCAAAGGATGGAGCTGTTTCAGGCGTTATTGATTGGTCAACAGCCCATATAGATGACCCAGCGATTGATTTTGCT  GGGCATGTAACTTTGTTTGGAGAAGAAAGCCTCAAAACTCTAATCATCGAGTATGAAAAACTAGGGGGTAAAGTTTGGAATAAACTATATGAACAGACTTTAGAAAGAGCAGCGGCCTAGATCGGAA | | No 100 % identity found  Highest percentage identity reported: 100 % Query Cover: 96 % | |  |
| Gene | Allele Sequence | | NCBI BLAST/CARD Result | | |
| *ere*A  ASV 1  ASV 2  ASV 3 | >consensus_1  CACGTTGATATGCTGACTCACTTGTTGGCGTCCATTGATGGCCAGTCGGCGGTTATTTCATCGGCAAAATGGGGGGAGCTAGAAACGGCTCGGCAGGAGAAAGCTATCTCAGGGGTAACCAGATTGAAGCTCCGCTTGGCGTCGCTTGCCCCTGTCCTGAAAAAACACGTCAACAGCGATTTGTTCCGAAAAGCCTCTGATCGAATAGAGTCGATAGAGTATACGTTGGAAACCTTGCGTATAATGAAAACTTTCTTCGATGGTACCTCTC | | *Providencia huaxiensis*  *Pandoraea sp.*  *Comamonas sp.*  *Vogesella fluminis*  *Uncultured bacterium*  *Vogesella perlucida*  *Comamonas koreensis* | |  |
|  | >consensus_2  CACGTTGATATGCTGACTCACTTGTTGGCGTCCATTGATGGCCAGTCGGCGGTTATTTCATCGGCAAAATGGGGGGAGCTAGAAACGGCTCGGCAGGAGAAAGCTATCTCAGGGGTAACCAGATTGAAGCTCCGCTTGGCATCGCTTGCCCCTGTCCTGAAAAAACACGTCAACAGCGATTTGTTCCGAAAAGCCTCTGATCGAATAGAGTCGATAGAGTATACGTTGGAAACCTTGCGTATAATGAAAACTTTCTTCGATGGTACCTCTC | | No 100% identity found  Highest percentage identity reported: 99.63 % Query Cover: 100 % | |  |
|  | >consensus_3  CACGTTGATATGTTGACTCACTTGTTGGCGTCCATTGATGGCCAGTCGGCGGTTATTTCATCGGCAAAATGGGGGGAGCTAGAAACGGCTCGGCAGGAGAAAGCTATCTCAGGGGTAACCAGATTGAAGCTCCGCTTGGCGTCGCTTGCCCCCGTCCTGAAAAAACACGTCAACAGCGATTTGTTCCGAAAAGCCTCTGATCGAATAGAGTCGATAGAGTATACGTTGGAAACCTTGCGTATAATGAAAACTTTCTTCGATGGTACCTCTC | | *Achromobacter denitrificans*  *Enterobacter hormaechei*  *Escherichia coli*  *Helicobacter pylori*  *Klebsiella pneumoniae*  *Pseudomonas aeruginosa*  *Salmonella enterica* | |  |
| ASV 4  Allele 5 | >consensus_4  CACGTTGATATGCTGACTCACTTGTTGGCGTCCATTGATGGCCAGTCGGCGGTTATTTCATCGGCAAAATGGGGGGAGCTAGAAACGGCTCGGCAGGAGAAAGCTATCTCAGGGGTAACCAGATTGAAGCTCCGCTTGGCGTCGCTTGCCCCTGTACTGAAAAAACACGTCAACAGCGATTTGTTCCGAAAAGCCTCTGATCGAATAGAGTCGATAGAGTATACGTTGGAAACCTTGCGTATAATGAAAACTTTCTTCGATGGTACCTCTC | | *Escherichia coli*  *Pseudomonas aeruginosa*  *Salmonella enterica*  *Serratia marcescens*  *Thauera humireducens*  *Uncultured bacterium* | |  |
| Gene | Allele Sequence | | NCBI BLAST/CARD Result | |  |
| *ere*A (continued)  ASV 5 | >consensus_5  CACGTTGATATGCTGACTCACTTGTTGGCGTCCATTGATGGCCAGTCGGCGGTTATTTCATCGGCAAAATGGGGGGAGCTAGAAACGGCTCGGCAGGAGAAAGCTATCTCAGGGGTAACCAGATTGAAGCTCCGCTTGGCGTCGCTTGCCCCTGTACTGAAAAAACACGTCAACAGCGATTTGTTCCGAAAAGCCTCTGATCGAATAGAATCGATAGAGTATACGTTGGAAACCTTGCGTATAATGAAAACTTTCTTCGATGGTACCTCTC | | *Aeromonas hydrophila*  *Escherichia coli*  *Klebsiella pneumoniae*  *Klebsiella oxytoca*  *Laribacter hongkongensis*  *Proteus mirabilis*  *Proteus terrae*  *Proteus vulgaris*  *Providencia rettgeri*  *Pseudoalteromonas sp.*  *Salmonella enterica*  *Vibrio alginolyticus*  *Vogesella perlucida* | |  |
| *rob*A  ASV 1  ASV 2 | >consensus_1  CACGATTTTCTCGGCAACGCGCCGACCATTCCGCCAGTGCTCTACGGCCTGAATGAAACGCGTCCGAGTCAGGATAAAGACGACGAACAAGAGGTATTCTATACCACCGCGTTAGCCCAGGATCAGGCAGATGGCTATGTACTGACGGGGCATCCGGTGATGCTGCAGGGCGGCGAATATGTGATGTTTACCTATGAAGGTCTGGGAACCGGCGTGCAGGAGTTTATCCTGACGGTATACGGAACGTGCATGCCAATGCTCAACCTGACGCGCC | | *Escherichia coli*  *Shigella dysenteriae*  *Shigella flexneri* | |  |
|  | >consensus_2  CGCGACTTCCTGAGCCATGCCCCGGCGATCCCGCCTATCCTGTATGGTCTCAACGAAACGCATCCAAGCCAGGAAAAAGACGACGAGCAGGAGGTGTTCTACACCACCGCGTTAACGCCGGAAATGGCCAATGGCTATATTCAGGGCTCTAAACCGGTCGTGCTGGAAGGCGGAGAGTACGTGATGTTCTCCTACGAAGGGCTGGGAACGGGCGTACAGGAATTCATCCTGACCGTTTACGGAACATGCATGCCTATGCTGAACCTGAATCGCC | | *Enterobacter cloacae* | |  |
| Gene | Allele Sequence | | NCBI BLAST/CARD Result | | |
| *rob*A (continued)  ASV 3  ASV 4  ASV 5 | >consensus_3  CGCGACTTCCTGAGCCACGCACCGGCGATCCCGCCTATTCTGTATGGTCTCAACGAAACGCACCCGAGCCAGGAAAAGGA  CGACGAGCAGGAGGTGTTCTACACCACCGCGCTGACGCCAGAGATGGCCAATGGCTACATTCAGGGTTCAAAACCTGTCG  TGCTGGAAGGCGGTGAATACGTGATGTTCGCCTATGAAGGGCTGGGAACGGGCGTTCAGGAGTTCATCCTGACCGTTTAC  GGAACCTGCATGCCGATGCTGAATCTGAATCGCC | | *Enterobacter cloacae* |  | |
|  | >consensus_4  CACGATTTTCTCGGCAACGCGCCGACCATTCCGCCAGTGCTCTACGGCCTGAATGAAACGCGTCCGAGTCAGGATAAAGA  CGACGAACAAGAGGTATTCTATACCACTGCGTTAGCCCAGGATCAGGCAGATGGCTATGTACTGACGGGGCATCCGGTGA  TGCTGCAGGGCGGCGAATATGTGATGTTTACCTATGAAGGTCTGGGAACCGGCGTGCAGGAGTTTATCCTGACAGTATAC  GGAACGTGCATGCCAATGCTCAATCTGACGCGCC | | *Escherichia coli* |  | |
|  | >consensus_5  GCACGATTTTCTCGGCAACGCGCCGACCATTCCGCCGGTGCTCTACGGCCTGAACGAAACGCGTCCGAGTCAGGATAAAG  ACGACGAACAAGAGGTATTCTATACCACCGCGTTAGCCCAGGATCAGGCAGATGGCTATGTACTGACGGGGCATCCGGTG  ATGCTGCAGGGCGGCGAATATGTGATGTTTACCTATGAAGGTCTGGGAACCGGCGTGCAGGAGTTTATCCTGACGGTATA  CGGAACGTGCATGCCAATGCTCAACCTGACGCGCC | | *Escherichia coli* |  | |
| Gene | Allele Sequence | | NCBI BLAST/CARD Result | | |
| *tet*O  ASV 1  ASV 2 | >consensus_1  TCCACTTTGAAATTTATGCACCGCAGGAATATCTCTCACGGGCGTATCATGATGCTCCAAGGTATTGTGCAGATATTGTA  AGTACTCAGATAAAGAATGACGAGGTCATTCTGAAAGGAGAAATCCCTGCTAGATGTATTCAAGAATACAGGAACGATTT  AACTAATTTCACAAATGGGCAGGGAGTCTGCTTGACAGAGTTAAAAGGATACCAGCCAGCTATTGGTAAATTTATTTGCC  AACCCCGCCGCCCGAATAGCCGTATAGATAAG | | *Bifidobacterium thermophilum*  *Campylobacter jejuni*  *Lactobacillus johnsonii*  *Riemerella anatipestifer*  *Streptococcus galloyticus*  *Streptococcus phage*  *Streptococcus suis*  *Uncultured bacterium* |  | |
|  | >consensus_2  TCCACTTTGAAATTTATGCACCGCAGGAATATCTCTCACGGGCGTATCATGATGCTCCAAGGTATTGTGCAGATATTGTA  AGTACTCAGATAAAGAATGACGAGGTCATTCTGAAAGGAGAAATCCCTGCTAGATGTATTCAAGAATACAGGAACGATTT  AACTTATTTCACAAATGGGCAGGGAGTCTGCTTGACAGAGTTAAAAGGATACCAGCCAGCTATTGGTAAATTTATTTGCC  AACCCCGCCGCCCGAATAGCCGTATAGATAAG | | *Actinobacillus pleuropneumoniae*  *Anaerostipes rhamnosivorans*  *Bifidobacterium breve*  *Bifidobacterium pseudocatenulatum*  *Blautia massiliensis*  *Blautia sp.*  *Campylobacter coli*  *Campylobacter jejuni*  *Clostridiales genomosp,*  *Clostridioides difficile*  *Coprococcus comes*  *Eggerthella lenta*  *Emergencia timonensis*  *Enterocloster bolteae*  *Enterocloster clostridioformis*  *Enterococcus cecorum*  *Enterococcus faecalis*  *Enterococcus gallinarum* | *Erysipelotrichaceae bacterium*  *Eubacterium sp.*  *Faecalibacterium prausnitzii*  *Glaesserella parasuis*  *Lachnospiraceae bacterium*  *Roseburia intestinalis*  *Streptococcus dysgalactiae*  *Streptococcus porcinus*  *Streptococcus pyogenes*  *Streptococcus suis*  *Uncultured Blautia sp.*  *Uncultured Dorea sp.*  *Uncultured Eubacteriales bacterium*  *Clostridium hylemonae*  *Clostridium innocuum*  *Clostridium scindens*  *Clostridium symbiosum* | |
